# Crystal structures of bis­[2-(pyridin-2-yl)phenyl-κ^2^ *N*,*C* ^1^]rhodium(III) complexes containing an aceto­nitrile or monodentate thyminate(1−) ligand

**DOI:** 10.1107/S2056989016004837

**Published:** 2016-03-24

**Authors:** Mika Sakate, Haruka Hosoda, Takayoshi Suzuki

**Affiliations:** a Department of Chemistry, Faculty of Science, Okayama University, Okayama 700-8530, Japan

**Keywords:** crystal structure, monodentate monoanionic thyminate, intra­molecular hydrogen-bonding inter­action, inter­molecular double hydrogen bonds

## Abstract

The crystal structures of bis­[2-(pyridin-2-yl)phen­yl]rhodium(III) complexes with the metal in an octahedral coordination containing chloride and aceto­nitrile ligands, namely (*OC*-6-42)-aceto­nitrile­chlorido­bis­[2-(pyridin-2-yl)phenyl-κ^2^ *N*,*C* ^1^]rhodium(III), [RhCl(C_11_H_8_N)_2_(CH_3_CN)] (**1**), thyminate(1−) and methanol, namely (*OC*-6-42)-methanol(5-methyl-2,4-dioxo-1,2,3,4-tetrahydro­pyrimidin-1-ido-κ*N* ^1^)bis­[2-(pyridin-2-yl)phenyl-κ^2^ *N*,*C* ^1^]rhodium(III), [Rh(C_11_H_8_N)_2_(C_5_H_5_N_2_O_2_)(CH_3_OH)]·CH_3_OH·0.5H_2_O (**2**), and thy­min­ate(1−) and ethanol, namely (*OC*-6-42)-ethanol(5-methyl-2,4-dioxo-1,2,3,4-tetra­hydro­pyrimidin-1-ido-κ*N* ^1^)bis[2-(pyridin-2-yl)phenyl-κ^2^ *N*,*C* ^1^]rhodium(III), [Rh(C_11_H_8_N)_2_(C_5_H_5_N_2_O_2_)(C_2_H_5_OH)]·C_2_H_5_OH (**3**), are reported. The aceto­nitrile complex, **1**, is isostructural with the Ir^III^ analog. In complexes **2** and **3**, the monodeprotonated thyminate (Hthym^−^) ligand coordinates to the Rh^III^ atom through the N atom, and the resulting Rh—N(Hthym) bond lengths are relatively long [2.261 (2) and 2.252 (2) Å for **2** and **3**, respectively] as compared to the Rh—N bonds in the related thyminate complexes. In each of the crystals of **2** and **3**, the complexes are linked *via* a pair of inter­molecular N—H⋯O hydrogen bonds between neighbouring Hthym^−^ ligands, forming an inversion dimer. A strong intra­molecular O—H⋯O hydrogen bond between the thyminate(1−) and alcohol ligands in mutually *cis* positions to each other is also observed.

## Abstract 2

Bis[2-(pyridin-2-yl)phen­yl]rhodium(III) complexes bearing aceto­nitrile or monodeprotonated thyminate (Hthym^−^) were characterized by X-ray analysis, and in the latter complexes it was revealed that Hthym^−^ coordinated to an Rh^III^ centre through the N^1^ atom together with a hydrogen-bonded methanol or ethanol co-ligand in the *cis* position.

## Chemical context   

Thymine (= H_2_thym) is one of the nucleobases, which are biologically important and fundamental organic mol­ecules, and can release one or two protons, giving a thyminate(1−) (= Hthym^−^) or thyminate(2−) (= thym^2–^) anion. These anions can act as suitable bridging ligands for the construction of functional polymetallic coordination compounds because they provide multiple donor atoms to metal atoms in a configurationally fixed fashion. For example, some tetra- and penta­nuclear Pt^II^ complexes bridged by thym^2–^ have been described (Khutia *et al.*, 2011 ▸; Rauterkus & Krebs, 2004 ▸). We have also reported some cyclic tetra­nuclear Cp*Rh^III^ (Cp* = penta­methyl­cyclo­penta­dien­yl) complexes bridged by thym^2–^ and incorporating an another metal cation in the central hydro­philic cavity of their metallacalix[4]arene motifs (Kashima *et al.*, 2015 ▸; Sakate *et al.*, 2016 ▸). In contrast, monoanionic thyminate (Hthym^−^) often acts as an N^1^-coordinating monodentate ligand, for example, in [{Cp*Rh(Hthym)}_2_(μ-OH)_2_] (Sakate *et al.*, 2016 ▸), [Cp*IrCl(Hthym)(dmso)] (dmso = di­methyl­sulfoxide; Krämer *et al.*, 1991 ▸), [Pt(NH_3_)_2_(Hthym)(Mecyto)]ClO_4_ (Mecyto = 1-meth­yl­cyto­sine; Faggiani *et al.*, 1981 ▸) and [(Tp^Cum,Me^)Zn(Hthym)]·EtAde {Tp^Cum,Me^ = hydrido­tris[2-methyl-4-(cumen-4-yl)-1-pyrazor­yl]borate, EtAde = 9-ethyl­adenine; Badura & Vahrenkamp, 2002 ▸}. The Zn^II^ complex is an inter­esting example, because the coordinating thyminato ligand forms multiple hydrogen bonds with the co-crystallized 9-ethyl­adenine mol­ecule.

Our next targets are cyclic polymetallic compounds built up with inter­molecular double hydrogen bonds between the coordinating thyminato(1−) and adeninato ligands. One of the complexes in this strategy is [Rh(ppy)_2_(Hthym)(ade)]^−^ [ppy^−^ = 2-(pyridin-2-yl)phenyl, ade^−^ = adeninato]. For this purpose, we have prepared stepwise from [Rh(ppy)_2_Cl(CH_3_CN)] (**1**), [Rh(ppy)_2_(Hthym)(CH_3_OH)]·CH_3_OH·0.5H_2_O (**2**) to [Rh(ppy)_2_(Hthym)(C_2_H_5_OH)]·C_2_H_5_OH (**3**), and have characterized their crystal structures. Attempts to react **2** or **3** with adenine or other monodentate ligands were also examined.
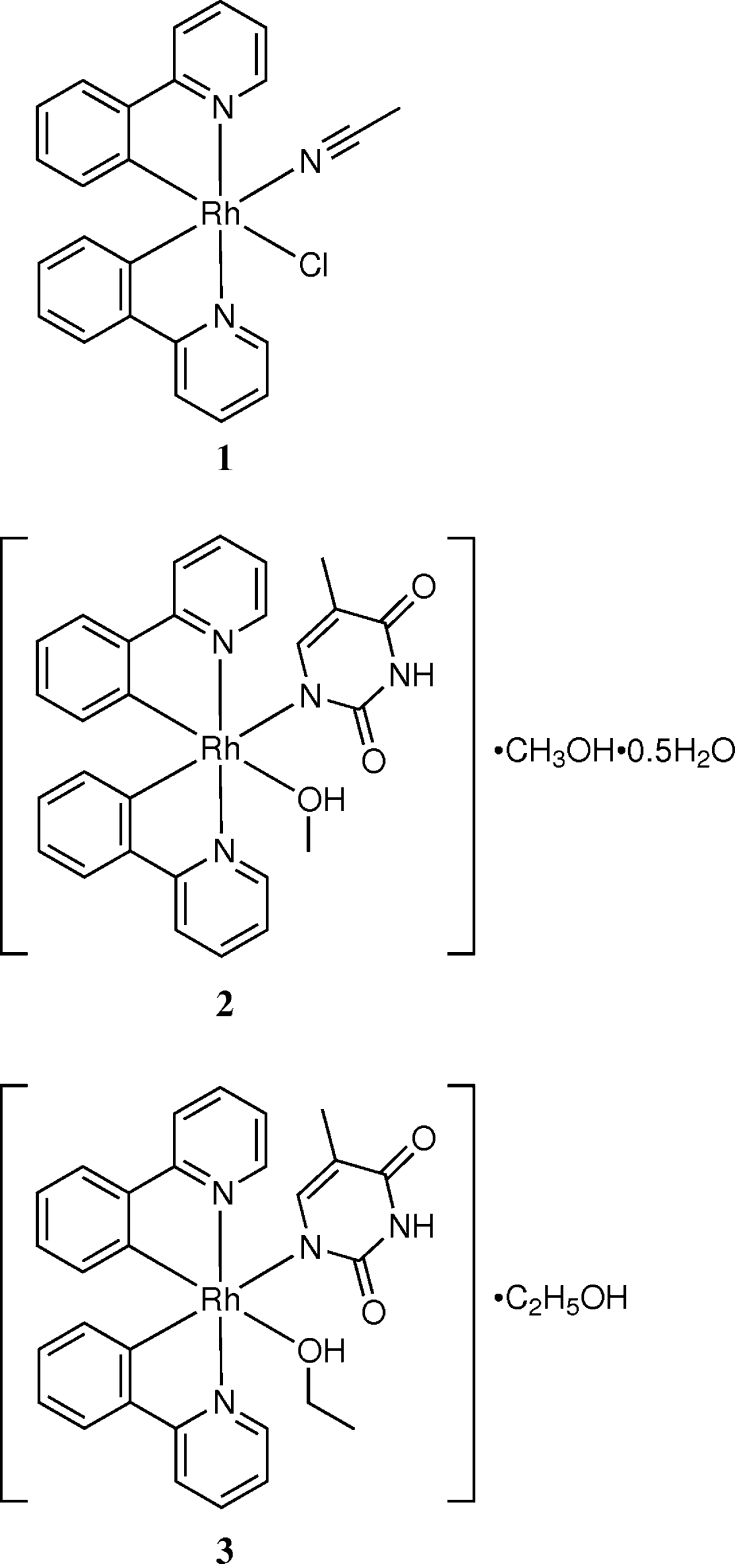


## Structural commentary   

**Figure 1:**
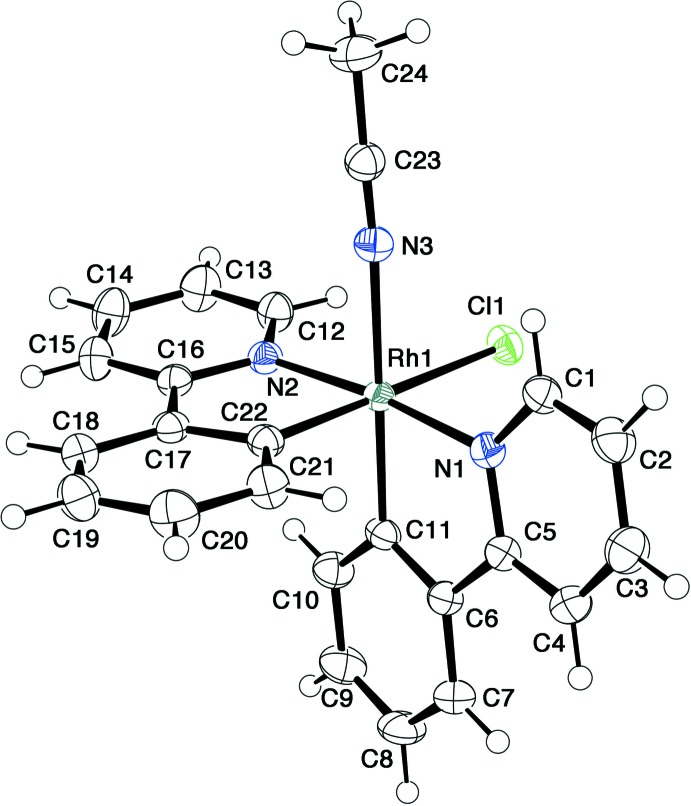
An *ORTEP* drawing of the mol­ecular structure of [Rh(ppy)_2_Cl(CH_3_CN)] (**1**), showing the atom-numbering scheme, with ellipsoids drawn at the 50% probability level.

Complexes **1**–**3** all have an octa­hedral coordination geometry with a *trans(N,N)cis(C,C)* configuration of the Rh^III^(ppy)_2_ fragment. The aceto­nitrile complex **1** (Fig. 1 ▸) is isostructural with the Ir^III^ analog, [Ir(ppy)_2_Cl(CH_3_CN)] (Blasberg *et al.*, 2011 ▸). The mutually *trans* Rh—N(ppy) bonds are 2.030 (1) and 2.051 (1) Å, and the *cis* Rh—C(ppy) bonds are almost the same as each other [1.990 (2) and 1.993 (2) Å]. The Rh—Cl and Rh—N(CH_3_CN) bonds in **1** are 2.4862 (4) and 2.162 (1) Å, respectively, which are almost at the longest end in the ranges of these bond lengths for the related Rh^III^ chlorido and aceto­nitrile complexes. These elongations are caused by the strong *trans* influence of the phenyl donor group. The aceto­nitrile mol­ecule is almost linearly coordin­ated, as evidenced by the bond angles Rh1—N3—C23 = 175.44 (14)° and N3—C23—C24 = 178.48 (19)°.

**Figure 2:**
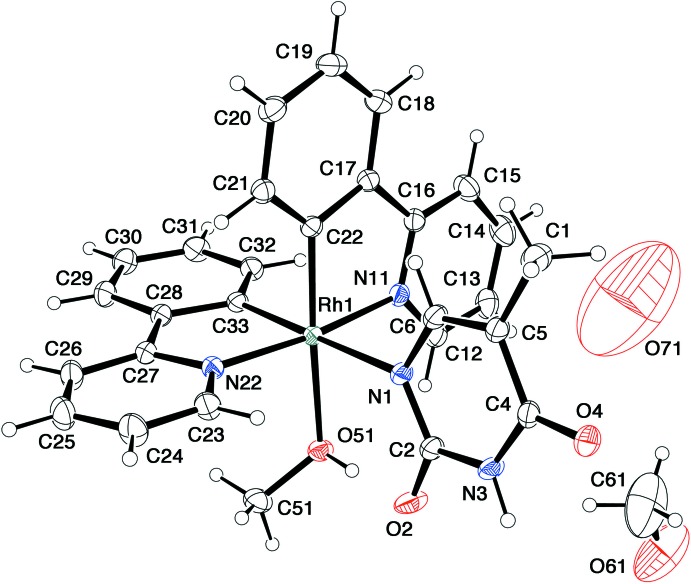
An *ORTEP* drawing of the mol­ecular structure of [Rh(ppy)(Hthym)(MeOH)]·MeOH·0.5H_2_O (**2**), showing the atom-numbering scheme, with ellipsoids drawn at the 30% probability level.

Crystals of **2** and **3** are solvatomorphs crystallizing in the space group *Pbca*, although the lengths of their *a* axes differ by more than 0.4 Å. In these complexes, the Hthym^−^ anion coordinates to the Rh^III^ atom as a monodentate ligand through the N^1^ atom (Figs. 2 ▸ and 3 ▸). There is a coordinating solvent (methanol or ethanol) mol­ecule in the *cis* position to the Hthym^−^ anion. The mutually *trans* Rh—N(ppy) bond lengths in **2** and **3** are in the range 2.023 (2)–2.038 (2) Å. On the other hand, the mutually *cis* Rh—C(ppy) bonds show explicit deviation; the Rh—C bonds *trans* to Hthym^−^ are 1.994 (2) and 1.989 (2) Å for **2** and **3**, respectively, while those *trans* to MeOH/EtOH in **2** and **3** are slightly shorter at 1.972 (2) and 1.976 (2) Å, respectively. The Rh—N(Hhtym) bonds in **2** and **3** are 2.261 (2) and 2.252 (2) Å, respectively, which are remarkably long as compared to those in the other Rh^III^–Hthym^−^ complexes. For example, the Rh—N(Hthym) bond in [{Cp*Rh(Hthym)}_2_(μ-OH)_2_] is 2.126 (3) Å (Sakate *et al.*, 2016 ▸). In the cyclic tetra­nuclear complexes bridged by thym^2–^, the Rh—N(thym^2–^) bonds are even shorter at 2.07 (1)–2.13 (1) Å. The Rh—O bonds in **2** and **3** are 2.233 (2) and 2.207 (1) Å, respectively, considerably longer than that [2.103 (3) Å] in [RhCl_3_(bpy)(CH_3_OH)] (Bieda *et al.*, 2009 ▸). However, much longer Rh—O(MeOH or EtOH) bonds (2.240 and 2.264 Å) are observed in *trans(C,O)-*[(PCP)RhCl_2_(MeOH or EtOH)] [PCP = 2,6-bis­(di­cyclo­hexyl­phosphinometh­yl)phenyl; Cross *et al.*, 1995 ▸]. These examples also indicate the strong *trans* influence of the phenyl-C donor in the *trans* position.

In both **2** and **3**, there is an intra­molecular hydrogen bond between atom O2 of the Hthym^−^ and O51—H1 of MeOH or EtOH in the mutually *cis*-position (Tables 2 and 3). These hydrogen bonds may stabilize the coordination of solvent MeOH and EtOH mol­ecules in **2** and **3**, even though the Rh—O bonds for these ligands are relatively long. In fact, a reaction of complex **2** or **3** with an equivalent amount of PPh_3_, P(OMe)_3_, imidazole or a mixture of adenine and tri­ethyl­amine (*L*) gave a complicated mixture of products, from which no desirable ligand-substituted complexes of the formula, [Rh(ppy)_2_(Hthym)(*L*)] could be isolated.

## Supra­molecular features   

In the crystal of the aceto­nitrile complex **1**, there are no remarkable inter­molecular hydrogen bonds. As similar to the Ir^III^ analog (Blasberg *et al.*, 2011 ▸), there are weak C—H⋯Cl hydrogen bonds (Table 1 ▸), which link the complexes into a layer parallel to the *bc* plane. In addition, C—H⋯π(ppy) [C8—H8⋯C16^iii^: H8⋯C16^iii^ = 2.81, C8⋯C16^iii^ = 3.620 (3) Å, C8—H8⋯C16^iii^ = 144°; symmetry code: (iii) *x* + 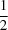, *y*, −*z* + 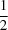] and C—H⋯π(nitrile) [C14—H14⋯C23^iv^: H14⋯C23^iv^ = 2.69, C14⋯C23^iv^ = 3.427 (2) Å, C14—H14⋯C23^iv^ = 135°; symmetry code: (iv) −*x*, *y* + 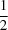, −*z* + 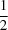] inter­actions are observed.

**Figure 4:**
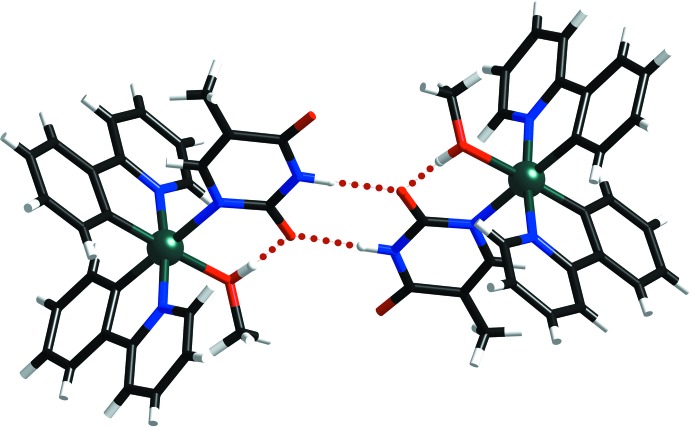
A perspective view of **2**, showing the intra- and inter­molecular O—H⋯O hydrogen bonds (dotted lines) between the Hthym^−^ and MeOH ligands.

In each crystal of the thyminato(1−) complexes of **2** and **3**, together with an intra­molecular hydrogen bond mentioned above, there is a pair of inter­molecular N—H⋯O hydrogen bonds (Tables 2 ▸ and 3 ▸) with an 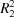
(8) ring motif between the neighboring Hthym^−^ ligands, forming an inversion dimer (Figs. 4 ▸ and 5 ▸). The methanol and ethanol mol­ecules of crystallization in **2** and **3** are each linked to the Hthym^−^ ligand *via* an inter­molecular O—H⋯O hydrogen bond.

## Synthesis and crystallization   

The starting rhodium(III) complex, [Rh(ppy)_2_Cl]_2_, was prepared by a literature method (Sprouse *et al.*, 1984 ▸). [Rh(ppy)_2_Cl]_2_ (0.050 g, 0.060 mmol) was dissolved in di­chloro­methane (5 mL) and aceto­nitrile (5 mL) was added to the solution. The mixture was allowed to stand in an open air to evaporate the solvent slowly, giving yellow crystals of **1**. Yield: 0.047 g (80%). Analysis found: C 58.64, H 3.65, N 8.49%. Calculated for C_24_H_19_ClN_3_Rh: C 59.09, H 3.93, N 8.61%.

To a methanol suspension (10 mL) of [Rh(ppy)_2_Cl]_2_ (0.090 g, 0.10 mmol) was added Ag(CF_3_SO_3_) (0.051 g, 0.20 mmol). The mixture was stirred at room temperature in the dark overnight, and the resulting white precipitate of AgCl was filtered off. A methanol solution (10 mL) containing thymine (0.025 g, 0.20 mmol) and tri­ethyl­amine (28 µL, 0.20 mmol) was carefully layered on the filtrate, and the mixture was allowed to stand overnight to give yellow crystals of **2**. Yield: 0.082 g (68%). Analysis found: C 58.05, H 4.62, N 9.30%. Calculated for C_29_H_30_N_4_O_4.5_Rh {= [Rh(ppy)_2_(Hthym)(CH_3_OH)]·CH_3_OH·0.5H_2_O}: C 57.15, H 4.96, N 9.19%. Complex **3** was prepared by a similar method to the above using ethanol as a solvent, instead of methanol. Yield: 64%. Analysis found: C 59.03, H 4.82, N 8.82%. Calculated for C_31_H_33_N_4_O_4_Rh: C 59.24, H 5.29, N 8.91%.

## Refinement   

Crystal data, data collection and structure refinement details are summarized in Table 4 ▸. All H atoms bonded to C and N atoms in **1**–**3** were refined using a riding model, with C—H = 0.95 or 0.98 Å and N—H = 0.88 Å, and with *U*_iso_(H) = 1.2*U*_eq_(C, N). The positions of the O-bound H atoms of the coordinating methanol mol­ecule in **2** and the coordinating and solvated ethanol mol­ecules in **3** were refined with the restraints O—H = 0.84 (1) Å, and with *U*_iso_(H) = 1.2*U*_eq_(O), while the H atom of the solvated methanol in **2** was refined using a riding model with O—H = 0.84 Å and *U*_iso_(H) = 1.2*U*_eq_(O). In the crystal of **2**, other than the complex and methanol mol­ecules, there is a small electron density remaining in the void, and this was assumed to be a water mol­ecule of crystallization. The H atoms of this water mol­ecule were not introduced in the calculation because of the highly disordered state of the water mol­ecule, which resulted in large thermal displacement parameters for the O atom.

## Supplementary Material

Crystal structure: contains datablock(s) 1, 2, 3, global. DOI: 10.1107/S2056989016004837/is5448sup1.cif

Structure factors: contains datablock(s) 1. DOI: 10.1107/S2056989016004837/is54481sup5.hkl

Structure factors: contains datablock(s) 2. DOI: 10.1107/S2056989016004837/is54482sup6.hkl

Structure factors: contains datablock(s) 3. DOI: 10.1107/S2056989016004837/is54483sup7.hkl

CCDC references: 1469935, 1469934, 1469933

Additional supporting information:  crystallographic information; 3D view; checkCIF report

## Figures and Tables

**Figure 3:**
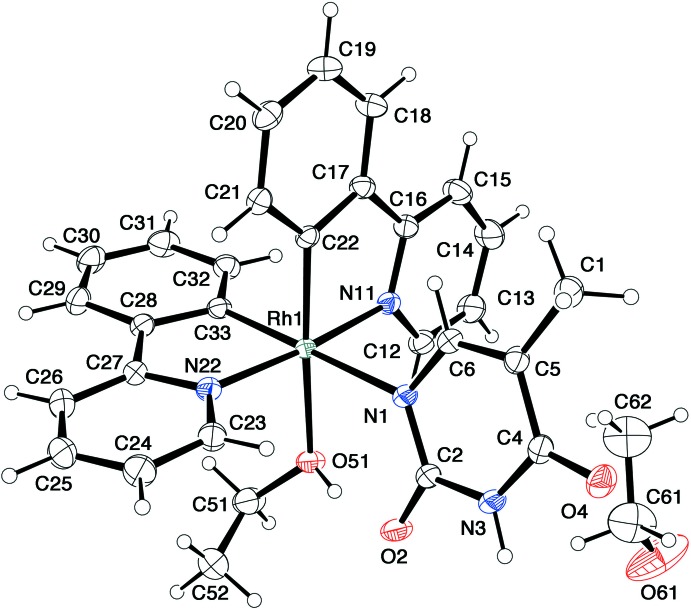
An *ORTEP* drawing of the mol­ecular structure of [Rh(ppy)(Hthym)(EtOH)]·EtOH (**3**), showing the atom-numbering scheme, with ellipsoids drawn at the 30% probability level.

**Figure 5:**
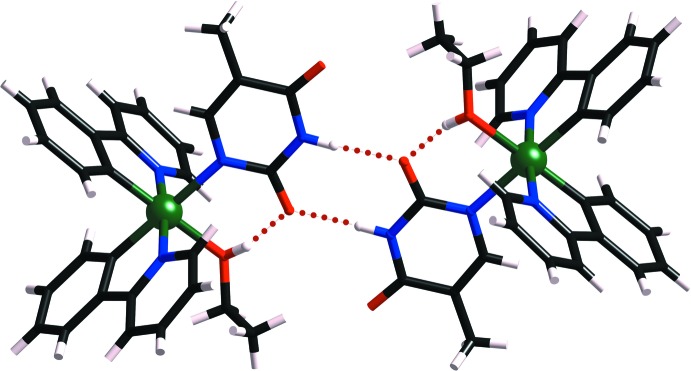
A perspective view of **3**, showing the intra- and inter­molecular O—H⋯O hydrogen-bonds (dotted lines) between the Hthym^−^ and EtOH ligands.

**Table 1:** Hydrogen-bond geometry (Å, °) for **1** [Chem scheme1]

*D*—H⋯*A*	*D*—H	H⋯*A*	*D*⋯*A*	*D*—H⋯*A*
C1—H1⋯Cl1^i^	0.95	2.79	3.613 (2)	145
C14—H14⋯Cl1^ii^	0.95	2.78	3.452 (2)	128

Symmetry codes: (i) 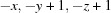; (ii) 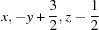.

**Table 2:** Hydrogen-bond geometry (Å, °) for **2** [Chem scheme1]

*D*—H⋯*A*	*D*—H	H⋯*A*	*D*⋯*A*	*D*—H⋯*A*
O51—H1⋯O2	0.84 (2)	1.69 (2)	2.527 (3)	170 (3)
N3—H3⋯O2^i^	0.88	1.97	2.844 (3)	173
O61—H2⋯O4^ii^	0.84	2.01	2.802 (5)	157

Symmetry codes: (i) 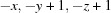; (ii) 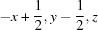.

**Table 3:** Hydrogen-bond geometry (Å, °) for **3** [Chem scheme1]

*D*—H⋯*A*	*D*—H	H⋯*A*	*D*⋯*A*	*D*—H⋯*A*
O51—H1⋯O2	0.83 (1)	1.72 (2)	2.527 (2)	164 (2)
N3—H3⋯O2^i^	0.88	1.99	2.854 (2)	165
O61—H2⋯O4^ii^	0.84 (1)	2.01 (4)	2.792 (3)	156 (4)

Symmetry codes: (i) 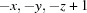; (ii) 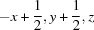.

**Table 4:** Experimental details

	**1**	**2**	**3**
Crystal data			
Chemical formula	[RhCl(C_11_H_8_N)_2_(C_2_H_3_N)]	[Rh(C_11_H_8_N)_2_(C_5_H_5_N_2_O_2_)(CH_4_O)]·CH_4_O·0.5H_2_O	[Rh(C_11_H_8_N)_2_(C_5_H_5_N_2_O_2_)(C_2_H_6_O)]·C_2_H_6_O
*M*_r_	487.78	609.48	628.52
Crystal system, space group	Orthorhombic, *P* *b* *c* *a*	Orthorhombic, *P* *b* *c* *a*	Orthorhombic, *P* *b* *c* *a*
Temperature (K)	193	192	192
*a*, *b*, *c* (Å)	16.5415 (9), 14.6600 (11), 17.0026 (12)	10.6964 (7), 15.5329 (9), 32.6325 (15)	11.1082 (5), 15.5556 (6), 32.6747 (15)
*V* (Å^3^)	4123.1 (5)	5421.8 (5)	5646.0 (4)
*Z*	8	8	8
Radiation type	Mo *K*α	Mo *K*α	Mo *K*α
μ (mm^−1^)	0.97	0.67	0.65
Crystal size (mm)	0.40 × 0.30 × 0.20	0.30 × 0.20 × 0.10	0.30 × 0.20 × 0.20

Data collection			
Diffractometer	Rigaku R-AXIS RAPID	Rigaku R-AXIS RAPID	Rigaku R-AXIS RAPID
Absorption correction	Numerical (*NUMABS*; Rigaku, 1999 ▸)	Numerical (*NUMABS*; Rigaku, 1999 ▸)	Numerical (*NUMABS*; Rigaku, 1999 ▸)
*T*_min_, *T*_max_	0.697, 0.829	0.824, 0.936	0.829, 0.881
No. of measured, independent and observed [*I* > 2σ(*I*)] reflections	38163, 4709, 4327	47794, 6196, 5307	52659, 6470, 5886
*R*_int_	0.042	0.046	0.030
(sin θ/λ)_max_ (Å^−1^)	0.649	0.649	0.649

Refinement			
*R*[*F* ^2^ > 2σ(*F* ^2^)], *wR*(*F* ^2^), *S*	0.024, 0.060, 1.07	0.034, 0.096, 1.04	0.029, 0.076, 1.07
No. of reflections	4709	6196	6470
No. of parameters	263	356	370
No. of restraints	0	1	2
H-atom treatment	H-atom parameters constrained	H atoms treated by a mixture of independent and constrained refinement	H atoms treated by a mixture of independent and constrained refinement
Δρ_max_, Δρ_min_ (e Å^−3^)	0.67, −0.35	1.29, −0.61	1.08, −0.40

Computer programs: *RAPID AUTO* (Rigaku, 2006 ▸), *CrystalStructure* (Rigaku, 2010 ▸), *Il Milione* (Burla *et al.*, 2007 ▸), *SHELXS2013* (Sheldrick, 2008 ▸), *SHELXL2013* (Sheldrick, 2015 ▸) and *ORTEP-3 for Windows* (Farrugia, 2012 ▸).

## supplementary crystallographic information

### (1) (OC-6-42)-Acetonitrilechloridobis[2-(pyridin-2-yl)phenyl-κ2N,C1]rhodium(III) Crystal data

**Table:**

[RhCl(C_11_H_8_N)_2_(C_2_H_3_N)]	*D*_x_ = 1.572 Mg m^−^^3^
*M**_r_* = 487.78	Mo *K*α radiation, λ = 0.71075 Å
Orthorhombic, *P**b**c**a*	Cell parameters from 31949 reflections
*a* = 16.5415 (9) Å	θ = 3.0–27.6°
*b* = 14.6600 (11) Å	µ = 0.97 mm^−^^1^
*c* = 17.0026 (12) Å	*T* = 193 K
*V* = 4123.1 (5) Å^3^	Block, yellow
*Z* = 8	0.40 × 0.30 × 0.20 mm
*F*(000) = 1968	

### (1) (OC-6-42)-Acetonitrilechloridobis[2-(pyridin-2-yl)phenyl-κ2N,C1]rhodium(III) Data collection

**Table:**

Rigaku R-AXIS RAPID diffractometer	4709 independent reflections
Radiation source: fine-focus sealed tube	4327 reflections with *I* > 2σ(*I*)
Graphite monochromator	*R*_int_ = 0.042
Detector resolution: 10.000 pixels mm^-1^	θ_max_ = 27.5°, θ_min_ = 3.0°
ω scans	*h* = −20→21
Absorption correction: numerical (NUMABS; Rigaku, 1999)	*k* = −18→18
*T*_min_ = 0.697, *T*_max_ = 0.829	*l* = −22→22
38163 measured reflections	

### (1) (OC-6-42)-Acetonitrilechloridobis[2-(pyridin-2-yl)phenyl-κ2N,C1]rhodium(III) Refinement

**Table:**

Refinement on *F*^2^	Primary atom site location: structure-invariant direct methods
Least-squares matrix: full	Secondary atom site location: difference Fourier map
*R*[*F*^2^ > 2σ(*F*^2^)] = 0.024	Hydrogen site location: inferred from neighbouring sites
*wR*(*F*^2^) = 0.060	H-atom parameters constrained
*S* = 1.07	*w* = 1/[σ^2^(*F*_o_^2^) + (0.0309*P*)^2^ + 1.6125*P*] where *P* = (*F*_o_^2^ + 2*F*_c_^2^)/3
4709 reflections	(Δ/σ)_max_ = 0.001
263 parameters	Δρ_max_ = 0.67 e Å^−^^3^
0 restraints	Δρ_min_ = −0.35 e Å^−^^3^

### (1) (OC-6-42)-Acetonitrilechloridobis[2-(pyridin-2-yl)phenyl-κ2N,C1]rhodium(III) Special details

**Table:**

Experimental. The ^1^H NMR spectrum of 1 in CD_2_Cl_2_ at 22 °C: δ 1.97 (s, 3H, MeCN), 5.90 (d, *J* = 7.8 Hz, 2H, ppy), 6.70 (td, *J* = 7.6 and 1.4 Hz, 2H, ppy), 6.77–6.96 (m, 4H, ppy), 7.62 (dd, *J* = 7.7 and 1.4 Hz, 2H, ppy), 7.81–7.98 (m, 4H, ppy) and 9.22 (d, *J* = 5.7 Hz, 2H, ppy).
Geometry. All esds (except the esd in the dihedral angle between two l.s. planes) are estimated using the full covariance matrix. The cell esds are taken into account individually in the estimation of esds in distances, angles and torsion angles; correlations between esds in cell parameters are only used when they are defined by crystal symmetry. An approximate (isotropic) treatment of cell esds is used for estimating esds involving l.s. planes.

### (1) (OC-6-42)-Acetonitrilechloridobis[2-(pyridin-2-yl)phenyl-κ2N,C1]rhodium(III) Fractional atomic coordinates and isotropic or equivalent isotropic displacement parameters (Å^2^)

**Table:**

	*x*	*y*	*z*	*U*_iso_*/*U*_eq_	
Rh1	0.12078 (2)	0.55701 (2)	0.36161 (2)	0.01986 (5)	
Cl1	0.10904 (2)	0.64708 (3)	0.48498 (2)	0.02906 (9)	
N1	0.15175 (8)	0.44472 (8)	0.42460 (8)	0.0238 (3)	
N2	0.09842 (8)	0.66466 (9)	0.28773 (8)	0.0235 (3)	
N3	−0.00853 (9)	0.53554 (10)	0.36314 (8)	0.0265 (3)	
C1	0.09933 (10)	0.38632 (11)	0.45823 (9)	0.0272 (3)	
H1	0.0433	0.4006	0.4579	0.033*	
C2	0.12459 (10)	0.30643 (13)	0.49304 (11)	0.0339 (4)	
H2	0.0865	0.2662	0.5165	0.041*	
C3	0.20657 (11)	0.28542 (12)	0.49338 (11)	0.0358 (4)	
H3	0.2251	0.2298	0.5157	0.043*	
C4	0.26051 (10)	0.34604 (12)	0.46102 (10)	0.0309 (3)	
H4	0.3167	0.3329	0.4617	0.037*	
C5	0.23289 (9)	0.42661 (11)	0.42724 (9)	0.0247 (3)	
C6	0.28290 (9)	0.49821 (11)	0.39221 (9)	0.0252 (3)	
C7	0.36733 (10)	0.49704 (13)	0.39354 (10)	0.0326 (4)	
H7	0.3954	0.4472	0.4166	0.039*	
C8	0.40989 (12)	0.56954 (14)	0.36084 (11)	0.0381 (4)	
H8	0.4673	0.5693	0.3614	0.046*	
C9	0.36859 (10)	0.64198 (13)	0.32751 (12)	0.0370 (4)	
H9	0.3979	0.6915	0.3054	0.044*	
C10	0.28439 (10)	0.64280 (12)	0.32621 (10)	0.0305 (3)	
H10	0.2568	0.6929	0.3031	0.037*	
C11	0.24040 (10)	0.57124 (11)	0.35831 (8)	0.0234 (3)	
C12	0.07628 (10)	0.74823 (11)	0.31092 (10)	0.0302 (3)	
H12	0.0715	0.7604	0.3656	0.036*	
C13	0.06014 (12)	0.81726 (12)	0.25807 (11)	0.0373 (4)	
H13	0.0435	0.8758	0.2758	0.045*	
C14	0.06879 (12)	0.79919 (12)	0.17872 (11)	0.0381 (4)	
H14	0.0591	0.8459	0.1411	0.046*	
C15	0.09143 (11)	0.71342 (12)	0.15440 (10)	0.0322 (4)	
H15	0.0978	0.7007	0.1000	0.039*	
C16	0.10502 (9)	0.64548 (11)	0.20997 (9)	0.0243 (3)	
C17	0.12356 (8)	0.54991 (11)	0.19292 (10)	0.0238 (3)	
C18	0.12867 (10)	0.51469 (13)	0.11672 (10)	0.0301 (4)	
H18	0.1222	0.5540	0.0728	0.036*	
C19	0.14314 (12)	0.42299 (13)	0.10501 (11)	0.0363 (4)	
H19	0.1462	0.3990	0.0532	0.044*	
C20	0.15317 (12)	0.36630 (13)	0.16931 (11)	0.0383 (4)	
H20	0.1636	0.3033	0.1614	0.046*	
C21	0.14813 (11)	0.40060 (12)	0.24553 (10)	0.0315 (3)	
H21	0.1555	0.3607	0.2890	0.038*	
C22	0.13243 (8)	0.49280 (11)	0.25894 (9)	0.0233 (3)	
C23	−0.07658 (11)	0.52843 (12)	0.36004 (9)	0.0285 (3)	
C24	−0.16431 (11)	0.51736 (16)	0.35764 (12)	0.0425 (5)	
H24A	−0.1903	0.5768	0.3653	0.051*	
H24B	−0.1813	0.4756	0.3995	0.051*	
H24C	−0.1802	0.4923	0.3065	0.051*	

### (1) (OC-6-42)-Acetonitrilechloridobis[2-(pyridin-2-yl)phenyl-κ2N,C1]rhodium(III) Atomic displacement parameters (Å^2^)

**Table:**

	*U*^11^	*U*^22^	*U*^33^	*U*^12^	*U*^13^	*U*^23^
Rh1	0.01770 (8)	0.02111 (8)	0.02077 (8)	−0.00061 (4)	0.00062 (4)	0.00068 (4)
Cl1	0.03143 (19)	0.0321 (2)	0.02369 (18)	−0.00410 (15)	0.00318 (15)	−0.00310 (15)
N1	0.0238 (7)	0.0248 (7)	0.0228 (6)	−0.0004 (5)	0.0003 (5)	0.0010 (5)
N2	0.0213 (6)	0.0243 (7)	0.0249 (6)	−0.0005 (5)	−0.0002 (5)	0.0017 (5)
N3	0.0238 (7)	0.0272 (7)	0.0286 (7)	−0.0010 (5)	0.0007 (5)	0.0000 (5)
C1	0.0245 (7)	0.0302 (8)	0.0268 (8)	−0.0028 (6)	0.0017 (6)	0.0020 (6)
C2	0.0350 (9)	0.0313 (9)	0.0355 (9)	−0.0043 (7)	0.0047 (7)	0.0069 (7)
C3	0.0401 (10)	0.0285 (9)	0.0386 (9)	0.0051 (7)	0.0012 (8)	0.0085 (7)
C4	0.0276 (8)	0.0321 (9)	0.0329 (8)	0.0057 (6)	0.0000 (7)	0.0029 (7)
C5	0.0231 (8)	0.0277 (8)	0.0233 (7)	0.0014 (6)	0.0011 (6)	−0.0003 (6)
C6	0.0220 (7)	0.0296 (8)	0.0241 (7)	0.0004 (6)	0.0009 (6)	−0.0008 (6)
C7	0.0225 (8)	0.0409 (10)	0.0345 (9)	0.0027 (7)	0.0017 (7)	0.0017 (8)
C8	0.0189 (9)	0.0473 (12)	0.0480 (12)	−0.0035 (7)	0.0055 (7)	0.0016 (8)
C9	0.0274 (8)	0.0365 (10)	0.0470 (11)	−0.0098 (7)	0.0076 (8)	0.0027 (8)
C10	0.0266 (8)	0.0292 (8)	0.0356 (9)	−0.0032 (6)	0.0024 (7)	0.0010 (7)
C11	0.0195 (7)	0.0275 (8)	0.0232 (7)	−0.0026 (6)	0.0028 (6)	−0.0028 (6)
C12	0.0353 (8)	0.0276 (8)	0.0275 (8)	0.0017 (7)	−0.0012 (7)	−0.0011 (6)
C13	0.0496 (11)	0.0245 (8)	0.0377 (9)	0.0061 (7)	−0.0038 (8)	0.0000 (7)
C14	0.0524 (11)	0.0283 (9)	0.0336 (9)	0.0017 (8)	−0.0068 (8)	0.0070 (7)
C15	0.0389 (10)	0.0328 (9)	0.0248 (8)	−0.0019 (7)	−0.0012 (7)	0.0041 (7)
C16	0.0200 (7)	0.0274 (8)	0.0254 (7)	−0.0016 (6)	−0.0008 (6)	0.0011 (6)
C17	0.0184 (7)	0.0268 (8)	0.0261 (8)	−0.0007 (5)	−0.0003 (6)	−0.0006 (6)
C18	0.0299 (8)	0.0361 (10)	0.0243 (8)	0.0018 (7)	0.0008 (6)	−0.0006 (7)
C19	0.0409 (10)	0.0388 (10)	0.0291 (9)	0.0022 (8)	0.0017 (8)	−0.0089 (7)
C20	0.0463 (11)	0.0282 (9)	0.0403 (10)	0.0030 (8)	0.0018 (9)	−0.0077 (7)
C21	0.0360 (9)	0.0275 (9)	0.0308 (8)	0.0009 (7)	0.0012 (7)	−0.0005 (7)
C22	0.0194 (7)	0.0260 (8)	0.0244 (7)	−0.0020 (5)	0.0010 (6)	−0.0015 (6)
C23	0.0276 (9)	0.0283 (9)	0.0296 (8)	−0.0005 (7)	0.0000 (6)	0.0006 (6)
C24	0.0220 (9)	0.0547 (13)	0.0507 (12)	−0.0032 (8)	−0.0031 (8)	0.0034 (9)

### (1) (OC-6-42)-Acetonitrilechloridobis[2-(pyridin-2-yl)phenyl-κ2N,C1]rhodium(III) Geometric parameters (Å, º)

**Table:**

Rh1—C11	1.9904 (16)	C9—H9	0.9500
Rh1—C22	1.9926 (15)	C10—C11	1.388 (2)
Rh1—N1	2.0296 (13)	C10—H10	0.9500
Rh1—N2	2.0507 (13)	C12—C13	1.379 (2)
Rh1—N3	2.1621 (14)	C12—H12	0.9500
Rh1—Cl1	2.4862 (4)	C13—C14	1.382 (3)
N1—C1	1.346 (2)	C13—H13	0.9500
N1—C5	1.369 (2)	C14—C15	1.376 (3)
N2—C12	1.338 (2)	C14—H14	0.9500
N2—C16	1.356 (2)	C15—C16	1.391 (2)
N3—C23	1.132 (2)	C15—H15	0.9500
C1—C2	1.377 (2)	C16—C17	1.463 (2)
C1—H1	0.9500	C17—C18	1.397 (2)
C2—C3	1.391 (2)	C17—C22	1.408 (2)
C2—H2	0.9500	C18—C19	1.380 (3)
C3—C4	1.374 (2)	C18—H18	0.9500
C3—H3	0.9500	C19—C20	1.383 (3)
C4—C5	1.391 (2)	C19—H19	0.9500
C4—H4	0.9500	C20—C21	1.393 (2)
C5—C6	1.463 (2)	C20—H20	0.9500
C6—C7	1.397 (2)	C21—C22	1.395 (2)
C6—C11	1.405 (2)	C21—H21	0.9500
C7—C8	1.391 (3)	C23—C24	1.461 (3)
C7—H7	0.9500	C24—H24A	0.9800
C8—C9	1.384 (3)	C24—H24B	0.9800
C8—H8	0.9500	C24—H24C	0.9800
C9—C10	1.393 (2)		
			
C11—Rh1—C22	85.91 (6)	C10—C9—H9	119.8
C11—Rh1—N1	81.31 (6)	C11—C10—C9	120.75 (17)
C22—Rh1—N1	93.13 (6)	C11—C10—H10	119.6
C11—Rh1—N2	94.67 (6)	C9—C10—H10	119.6
C22—Rh1—N2	81.05 (6)	C10—C11—C6	118.35 (15)
N1—Rh1—N2	173.19 (5)	C10—C11—Rh1	127.68 (13)
C11—Rh1—N3	177.47 (6)	C6—C11—Rh1	113.96 (11)
C22—Rh1—N3	92.15 (5)	N2—C12—C13	122.19 (16)
N1—Rh1—N3	97.20 (5)	N2—C12—H12	118.9
N2—Rh1—N3	86.62 (5)	C13—C12—H12	118.9
C11—Rh1—Cl1	92.62 (4)	C12—C13—C14	118.37 (16)
C22—Rh1—Cl1	176.01 (5)	C12—C13—H13	120.8
N1—Rh1—Cl1	90.30 (4)	C14—C13—H13	120.8
N2—Rh1—Cl1	95.40 (4)	C15—C14—C13	119.78 (16)
N3—Rh1—Cl1	89.42 (4)	C15—C14—H14	120.1
C1—N1—C5	119.62 (14)	C13—C14—H14	120.1
C1—N1—Rh1	125.26 (11)	C14—C15—C16	119.62 (16)
C5—N1—Rh1	114.97 (10)	C14—C15—H15	120.2
C12—N2—C16	119.94 (14)	C16—C15—H15	120.2
C12—N2—Rh1	124.99 (11)	N2—C16—C15	120.05 (15)
C16—N2—Rh1	115.03 (11)	N2—C16—C17	114.12 (14)
C23—N3—Rh1	175.44 (14)	C15—C16—C17	125.79 (15)
N1—C1—C2	121.87 (15)	C18—C17—C22	120.88 (15)
N1—C1—H1	119.1	C18—C17—C16	123.39 (15)
C2—C1—H1	119.1	C22—C17—C16	115.67 (15)
C1—C2—C3	119.09 (16)	C19—C18—C17	120.29 (17)
C1—C2—H2	120.5	C19—C18—H18	119.9
C3—C2—H2	120.5	C17—C18—H18	119.9
C4—C3—C2	119.22 (16)	C18—C19—C20	119.47 (17)
C4—C3—H3	120.4	C18—C19—H19	120.3
C2—C3—H3	120.4	C20—C19—H19	120.3
C3—C4—C5	120.08 (15)	C19—C20—C21	120.76 (17)
C3—C4—H4	120.0	C19—C20—H20	119.6
C5—C4—H4	120.0	C21—C20—H20	119.6
N1—C5—C4	120.04 (15)	C20—C21—C22	120.86 (16)
N1—C5—C6	113.69 (14)	C20—C21—H21	119.6
C4—C5—C6	126.28 (15)	C22—C21—H21	119.6
C7—C6—C11	121.09 (15)	C21—C22—C17	117.72 (14)
C7—C6—C5	123.36 (15)	C21—C22—Rh1	128.23 (12)
C11—C6—C5	115.52 (14)	C17—C22—Rh1	114.05 (12)
C8—C7—C6	119.36 (17)	N3—C23—C24	178.48 (19)
C8—C7—H7	120.3	C23—C24—H24A	109.5
C6—C7—H7	120.3	C23—C24—H24B	109.5
C9—C8—C7	120.01 (17)	H24A—C24—H24B	109.5
C9—C8—H8	120.0	C23—C24—H24C	109.5
C7—C8—H8	120.0	H24A—C24—H24C	109.5
C8—C9—C10	120.43 (17)	H24B—C24—H24C	109.5
C8—C9—H9	119.8		
			
C5—N1—C1—C2	−2.5 (2)	C16—N2—C12—C13	−0.5 (2)
Rh1—N1—C1—C2	173.05 (13)	Rh1—N2—C12—C13	−178.34 (13)
N1—C1—C2—C3	−0.1 (3)	N2—C12—C13—C14	−1.2 (3)
C1—C2—C3—C4	1.9 (3)	C12—C13—C14—C15	1.3 (3)
C2—C3—C4—C5	−1.1 (3)	C13—C14—C15—C16	0.3 (3)
C1—N1—C5—C4	3.2 (2)	C12—N2—C16—C15	2.2 (2)
Rh1—N1—C5—C4	−172.74 (12)	Rh1—N2—C16—C15	−179.80 (12)
C1—N1—C5—C6	−176.83 (14)	C12—N2—C16—C17	−175.52 (14)
Rh1—N1—C5—C6	7.22 (17)	Rh1—N2—C16—C17	2.54 (16)
C3—C4—C5—N1	−1.5 (2)	C14—C15—C16—N2	−2.1 (3)
C3—C4—C5—C6	178.60 (16)	C14—C15—C16—C17	175.30 (16)
N1—C5—C6—C7	175.24 (15)	N2—C16—C17—C18	176.48 (14)
C4—C5—C6—C7	−4.8 (3)	C15—C16—C17—C18	−1.0 (2)
N1—C5—C6—C11	−3.0 (2)	N2—C16—C17—C22	−0.54 (19)
C4—C5—C6—C11	176.96 (16)	C15—C16—C17—C22	−178.06 (15)
C11—C6—C7—C8	0.1 (3)	C22—C17—C18—C19	−0.5 (2)
C5—C6—C7—C8	−178.03 (16)	C16—C17—C18—C19	−177.33 (15)
C6—C7—C8—C9	0.1 (3)	C17—C18—C19—C20	−0.5 (3)
C7—C8—C9—C10	−0.2 (3)	C18—C19—C20—C21	0.6 (3)
C8—C9—C10—C11	0.1 (3)	C19—C20—C21—C22	0.3 (3)
C9—C10—C11—C6	0.1 (2)	C20—C21—C22—C17	−1.3 (2)
C9—C10—C11—Rh1	−178.98 (14)	C20—C21—C22—Rh1	178.95 (14)
C7—C6—C11—C10	−0.2 (2)	C18—C17—C22—C21	1.3 (2)
C5—C6—C11—C10	178.05 (14)	C16—C17—C22—C21	178.43 (14)
C7—C6—C11—Rh1	179.01 (13)	C18—C17—C22—Rh1	−178.86 (11)
C5—C6—C11—Rh1	−2.72 (17)	C16—C17—C22—Rh1	−1.75 (16)

### (1) (OC-6-42)-Acetonitrilechloridobis[2-(pyridin-2-yl)phenyl-κ2N,C1]rhodium(III) Hydrogen-bond geometry (Å, º)

**Table:**

*D*—H···*A*	*D*—H	H···*A*	*D*···*A*	*D*—H···*A*
C1—H1···Cl1^i^	0.95	2.79	3.613 (2)	145
C14—H14···Cl1^ii^	0.95	2.78	3.452 (2)	128

Symmetry codes: (i) −*x*, −*y*+1, −*z*+1; (ii) *x*, −*y*+3/2, *z*−1/2.

### (2) (OC-6-42)-Methanol(5-methyl-2,4-dioxo-1,2,3,4-tetrahydropyrimidin-1-ido-κN1)bis[2-(pyridin-2-yl)phenyl-κ2N,C1]rhodium(III) Crystal data

**Table:**

[Rh(C_11_H_8_N)_2_(C_5_H_5_N_2_O_2_)(CH_4_O)]·CH_4_O·0.5H_2_O	*D*_x_ = 1.493 Mg m^−^^3^
*M**_r_* = 609.48	Mo *K*α radiation, λ = 0.71075 Å
Orthorhombic, *P**b**c**a*	Cell parameters from 34894 reflections
*a* = 10.6964 (7) Å	θ = 3.1–27.5°
*b* = 15.5329 (9) Å	µ = 0.67 mm^−^^1^
*c* = 32.6325 (15) Å	*T* = 192 K
*V* = 5421.8 (5) Å^3^	Block, yellow
*Z* = 8	0.30 × 0.20 × 0.10 mm
*F*(000) = 2504	

### (2) (OC-6-42)-Methanol(5-methyl-2,4-dioxo-1,2,3,4-tetrahydropyrimidin-1-ido-κN1)bis[2-(pyridin-2-yl)phenyl-κ2N,C1]rhodium(III) Data collection

**Table:**

Rigaku R-AXIS RAPID diffractometer	6196 independent reflections
Radiation source: fine-focus sealed tube	5307 reflections with *I* > 2σ(*I*)
Graphite monochromator	*R*_int_ = 0.046
Detector resolution: 10.000 pixels mm^-1^	θ_max_ = 27.5°, θ_min_ = 3.1°
ω scans	*h* = −13→13
Absorption correction: numerical (NUMABS; Rigaku, 1999)	*k* = −20→20
*T*_min_ = 0.824, *T*_max_ = 0.936	*l* = −42→40
47794 measured reflections	

### (2) (OC-6-42)-Methanol(5-methyl-2,4-dioxo-1,2,3,4-tetrahydropyrimidin-1-ido-κN1)bis[2-(pyridin-2-yl)phenyl-κ2N,C1]rhodium(III) Refinement

**Table:**

Refinement on *F*^2^	Primary atom site location: structure-invariant direct methods
Least-squares matrix: full	Secondary atom site location: difference Fourier map
*R*[*F*^2^ > 2σ(*F*^2^)] = 0.034	Hydrogen site location: inferred from neighbouring sites
*wR*(*F*^2^) = 0.096	H atoms treated by a mixture of independent and constrained refinement
*S* = 1.04	*w* = 1/[σ^2^(*F*_o_^2^) + (0.0469*P*)^2^ + 5.8148*P*] where *P* = (*F*_o_^2^ + 2*F*_c_^2^)/3
6196 reflections	(Δ/σ)_max_ = 0.001
356 parameters	Δρ_max_ = 1.29 e Å^−^^3^
1 restraint	Δρ_min_ = −0.61 e Å^−^^3^

### (2) (OC-6-42)-Methanol(5-methyl-2,4-dioxo-1,2,3,4-tetrahydropyrimidin-1-ido-κN1)bis[2-(pyridin-2-yl)phenyl-κ2N,C1]rhodium(III) Special details

**Table:**

Experimental. The ^1^H NMR spectrum of 2 in CDCl_3_ at 22 °C: δ 1.50 (s, 3H, Hthym C*H*_3_), 6.17 (d, *J* = 7.7 Hz, 2H, ppy), 6.40 (s, 1H, Hthym C^6^-*H*), 6.81 (t, *J* = 7.3 Hz, 2H ppy), 6.94 (t, *J* = 7.6 Hz, 2H, ppy), 7.28–7.31 (m, 2H, ppy), 7.58–7.60 (m, 2H, ppy), 7.87–7.91 (m, 4H, ppy), 8.35 (s, 1H, Hthym N^3^-*H*), 8.59 (d, *J* = 5.3 Hz, 1H ppy) and 8.99 (d, *J* = 5.3 Hz, 1H, ppy).
Geometry. All esds (except the esd in the dihedral angle between two l.s. planes) are estimated using the full covariance matrix. The cell esds are taken into account individually in the estimation of esds in distances, angles and torsion angles; correlations between esds in cell parameters are only used when they are defined by crystal symmetry. An approximate (isotropic) treatment of cell esds is used for estimating esds involving l.s. planes.

### (2) (OC-6-42)-Methanol(5-methyl-2,4-dioxo-1,2,3,4-tetrahydropyrimidin-1-ido-κN1)bis[2-(pyridin-2-yl)phenyl-κ2N,C1]rhodium(III) Fractional atomic coordinates and isotropic or equivalent isotropic displacement parameters (Å^2^)

**Table:**

	*x*	*y*	*z*	*U*_iso_*/*U*_eq_	
Rh1	0.21119 (2)	0.49429 (2)	0.36187 (2)	0.02250 (7)	
O2	0.0485 (2)	0.47720 (13)	0.45127 (6)	0.0465 (5)	
O4	0.18704 (18)	0.69252 (12)	0.53187 (5)	0.0375 (4)	
O61	0.2102 (6)	0.3378 (3)	0.57003 (13)	0.1420 (19)	
H2	0.2587	0.2984	0.5624	0.170*	
O71	0.5000	0.5000	0.5000	0.523 (18)	
O51	0.07868 (16)	0.39468 (11)	0.38528 (5)	0.0320 (4)	
H1	0.067 (3)	0.4167 (17)	0.4086 (5)	0.038*	
N1	0.19674 (18)	0.56450 (13)	0.42240 (6)	0.0267 (4)	
N3	0.12027 (19)	0.58796 (14)	0.48895 (6)	0.0321 (4)	
H3	0.0659	0.5725	0.5078	0.039*	
N11	0.36388 (18)	0.43094 (12)	0.38330 (6)	0.0272 (4)	
N22	0.06810 (18)	0.55596 (13)	0.33315 (6)	0.0275 (4)	
C1	0.3749 (3)	0.74950 (19)	0.47238 (8)	0.0464 (7)	
H1A	0.4254	0.7569	0.4476	0.056*	
H1B	0.4295	0.7349	0.4955	0.056*	
H1C	0.3303	0.8032	0.4784	0.056*	
C2	0.1210 (2)	0.54094 (16)	0.45317 (7)	0.0301 (5)	
C4	0.1962 (2)	0.65651 (15)	0.49803 (7)	0.0286 (5)	
C5	0.2822 (2)	0.67846 (16)	0.46584 (7)	0.0301 (5)	
C6	0.2756 (2)	0.63197 (16)	0.43056 (7)	0.0306 (5)	
H6	0.3316	0.6480	0.4093	0.037*	
C12	0.3605 (3)	0.35579 (16)	0.40370 (8)	0.0339 (5)	
H12	0.2819	0.3308	0.4102	0.041*	
C13	0.4688 (3)	0.31390 (18)	0.41551 (9)	0.0432 (6)	
H13	0.4647	0.2609	0.4300	0.052*	
C14	0.5827 (3)	0.3501 (2)	0.40600 (10)	0.0485 (7)	
H14	0.6581	0.3224	0.4139	0.058*	
C15	0.5860 (3)	0.42733 (18)	0.38490 (9)	0.0417 (6)	
H15	0.6640	0.4526	0.3779	0.050*	
C16	0.4754 (2)	0.46771 (16)	0.37397 (7)	0.0292 (5)	
C17	0.4635 (2)	0.54941 (15)	0.35162 (7)	0.0275 (5)	
C18	0.5653 (2)	0.60157 (17)	0.34154 (8)	0.0367 (6)	
H18	0.6478	0.5839	0.3483	0.044*	
C19	0.5459 (3)	0.67878 (18)	0.32166 (9)	0.0410 (6)	
H19	0.6150	0.7144	0.3148	0.049*	
C20	0.4252 (2)	0.70452 (17)	0.31173 (8)	0.0368 (6)	
H20	0.4119	0.7578	0.2981	0.044*	
C21	0.3236 (2)	0.65245 (16)	0.32164 (7)	0.0299 (5)	
H21	0.2416	0.6705	0.3145	0.036*	
C22	0.3404 (2)	0.57451 (15)	0.34184 (6)	0.0254 (4)	
C23	−0.0065 (2)	0.61523 (17)	0.35045 (8)	0.0354 (5)	
H23	0.0084	0.6314	0.3781	0.042*	
C24	−0.1031 (3)	0.65325 (19)	0.32980 (9)	0.0440 (7)	
H24	−0.1535	0.6956	0.3428	0.053*	
C25	−0.1262 (3)	0.6288 (2)	0.28947 (9)	0.0465 (7)	
H25	−0.1927	0.6542	0.2745	0.056*	
C26	−0.0512 (2)	0.56704 (18)	0.27151 (8)	0.0383 (6)	
H26	−0.0667	0.5491	0.2442	0.046*	
C27	0.0469 (2)	0.53132 (16)	0.29358 (7)	0.0288 (5)	
C28	0.1342 (2)	0.46567 (16)	0.27854 (7)	0.0282 (5)	
C29	0.1326 (3)	0.43303 (17)	0.23851 (7)	0.0359 (5)	
H29	0.0719	0.4527	0.2195	0.043*	
C30	0.2196 (3)	0.37214 (19)	0.22686 (8)	0.0401 (6)	
H30	0.2188	0.3498	0.1998	0.048*	
C31	0.3081 (3)	0.34358 (17)	0.25468 (8)	0.0381 (6)	
H31	0.3680	0.3018	0.2465	0.046*	
C32	0.3101 (2)	0.37542 (16)	0.29443 (8)	0.0321 (5)	
H32	0.3710	0.3548	0.3132	0.039*	
C33	0.2237 (2)	0.43744 (15)	0.30723 (7)	0.0263 (5)	
C51	−0.0423 (3)	0.3826 (2)	0.36788 (9)	0.0435 (7)	
H51A	−0.0836	0.3338	0.3813	0.052*	
H51B	−0.0341	0.3709	0.3385	0.052*	
H51C	−0.0923	0.4348	0.3719	0.052*	
C61	0.2442 (8)	0.4080 (4)	0.55466 (19)	0.136 (3)	
H61A	0.3340	0.4059	0.5488	0.164*	
H61B	0.2271	0.4548	0.5740	0.164*	
H61C	0.1981	0.4181	0.5292	0.164*	

### (2) (OC-6-42)-Methanol(5-methyl-2,4-dioxo-1,2,3,4-tetrahydropyrimidin-1-ido-κN1)bis[2-(pyridin-2-yl)phenyl-κ2N,C1]rhodium(III) Atomic displacement parameters (Å^2^)

**Table:**

	*U*^11^	*U*^22^	*U*^33^	*U*^12^	*U*^13^	*U*^23^
Rh1	0.02134 (11)	0.02767 (11)	0.01849 (11)	0.00008 (6)	0.00094 (6)	−0.00116 (6)
O2	0.0542 (13)	0.0539 (11)	0.0314 (10)	−0.0286 (10)	0.0183 (9)	−0.0145 (9)
O4	0.0430 (10)	0.0444 (10)	0.0252 (8)	−0.0030 (8)	0.0005 (7)	−0.0095 (7)
O61	0.255 (6)	0.082 (2)	0.089 (3)	0.020 (3)	0.047 (3)	−0.015 (2)
O71	0.76 (5)	0.48 (3)	0.33 (2)	0.23 (3)	0.14 (3)	0.003 (18)
O51	0.0315 (9)	0.0361 (9)	0.0283 (8)	−0.0060 (7)	0.0054 (7)	−0.0050 (7)
N1	0.0281 (10)	0.0318 (10)	0.0200 (9)	−0.0024 (8)	0.0025 (7)	−0.0021 (8)
N3	0.0340 (11)	0.0420 (11)	0.0203 (9)	−0.0089 (9)	0.0083 (8)	−0.0063 (8)
N11	0.0283 (10)	0.0317 (10)	0.0215 (9)	0.0022 (8)	−0.0007 (7)	−0.0006 (8)
N22	0.0238 (10)	0.0340 (10)	0.0247 (9)	0.0013 (8)	−0.0003 (7)	−0.0008 (8)
C1	0.0575 (18)	0.0505 (16)	0.0314 (13)	−0.0242 (15)	0.0039 (12)	−0.0054 (12)
C2	0.0311 (12)	0.0364 (12)	0.0227 (11)	−0.0044 (10)	0.0033 (9)	−0.0043 (9)
C4	0.0305 (12)	0.0333 (12)	0.0219 (11)	0.0024 (9)	−0.0027 (9)	−0.0008 (9)
C5	0.0341 (13)	0.0331 (12)	0.0231 (11)	−0.0045 (10)	−0.0006 (9)	−0.0001 (9)
C6	0.0343 (13)	0.0352 (12)	0.0222 (11)	−0.0048 (10)	0.0027 (9)	−0.0004 (9)
C12	0.0377 (14)	0.0323 (12)	0.0318 (12)	−0.0002 (10)	−0.0025 (10)	0.0038 (10)
C13	0.0503 (17)	0.0382 (14)	0.0412 (15)	0.0050 (12)	−0.0116 (12)	0.0081 (12)
C14	0.0406 (16)	0.0465 (16)	0.0583 (18)	0.0106 (13)	−0.0144 (13)	0.0058 (14)
C15	0.0286 (13)	0.0460 (15)	0.0503 (16)	0.0023 (11)	−0.0068 (11)	0.0039 (13)
C16	0.0275 (12)	0.0352 (12)	0.0250 (11)	0.0010 (10)	−0.0013 (9)	−0.0008 (10)
C17	0.0256 (11)	0.0324 (12)	0.0244 (11)	0.0005 (9)	0.0014 (9)	−0.0004 (9)
C18	0.0255 (12)	0.0438 (14)	0.0408 (14)	−0.0017 (10)	0.0021 (10)	0.0027 (11)
C19	0.0325 (14)	0.0440 (15)	0.0464 (15)	−0.0086 (11)	0.0056 (11)	0.0062 (12)
C20	0.0405 (15)	0.0347 (13)	0.0354 (13)	−0.0019 (11)	0.0028 (11)	0.0070 (10)
C21	0.0286 (12)	0.0344 (12)	0.0265 (11)	0.0027 (9)	0.0013 (9)	0.0002 (9)
C22	0.0248 (11)	0.0315 (11)	0.0200 (10)	−0.0009 (9)	0.0034 (8)	−0.0039 (9)
C23	0.0319 (13)	0.0420 (14)	0.0323 (12)	0.0057 (11)	0.0005 (10)	−0.0068 (11)
C24	0.0374 (15)	0.0517 (16)	0.0429 (15)	0.0159 (12)	−0.0012 (12)	−0.0071 (13)
C25	0.0372 (15)	0.0627 (18)	0.0395 (15)	0.0171 (13)	−0.0062 (12)	0.0025 (13)
C26	0.0359 (14)	0.0506 (15)	0.0284 (12)	0.0075 (12)	−0.0061 (10)	−0.0017 (11)
C27	0.0263 (12)	0.0368 (12)	0.0233 (11)	−0.0005 (10)	−0.0002 (9)	0.0014 (9)
C28	0.0280 (12)	0.0346 (12)	0.0219 (10)	−0.0010 (9)	0.0002 (9)	−0.0015 (9)
C29	0.0371 (14)	0.0451 (14)	0.0255 (12)	−0.0014 (11)	−0.0029 (10)	−0.0048 (10)
C30	0.0437 (16)	0.0508 (16)	0.0257 (12)	−0.0038 (12)	0.0023 (10)	−0.0116 (11)
C31	0.0372 (14)	0.0406 (13)	0.0366 (14)	0.0035 (11)	0.0067 (11)	−0.0123 (11)
C32	0.0289 (12)	0.0368 (13)	0.0306 (12)	0.0020 (10)	0.0005 (9)	−0.0048 (10)
C33	0.0258 (12)	0.0307 (11)	0.0223 (10)	−0.0036 (9)	0.0019 (8)	−0.0020 (9)
C51	0.0346 (15)	0.0534 (17)	0.0424 (15)	−0.0136 (13)	0.0015 (11)	−0.0088 (13)
C61	0.243 (8)	0.070 (3)	0.095 (4)	−0.039 (4)	−0.041 (5)	−0.015 (3)

### (2) (OC-6-42)-Methanol(5-methyl-2,4-dioxo-1,2,3,4-tetrahydropyrimidin-1-ido-κN1)bis[2-(pyridin-2-yl)phenyl-κ2N,C1]rhodium(III) Geometric parameters (Å, º)

**Table:**

Rh1—C22	1.972 (2)	C16—C17	1.469 (3)
Rh1—C33	1.994 (2)	C17—C18	1.397 (3)
Rh1—N11	2.0309 (19)	C17—C22	1.410 (3)
Rh1—N22	2.0344 (19)	C18—C19	1.379 (4)
Rh1—O51	2.2331 (17)	C18—H18	0.9500
Rh1—N1	2.2614 (19)	C19—C20	1.390 (4)
O2—C2	1.259 (3)	C19—H19	0.9500
O4—C4	1.242 (3)	C20—C21	1.393 (3)
O61—C61	1.255 (6)	C20—H20	0.9500
O61—H2	0.8400	C21—C22	1.390 (3)
O51—C51	1.425 (3)	C21—H21	0.9500
O51—H1	0.844 (10)	C23—C24	1.368 (4)
N1—C2	1.341 (3)	C23—H23	0.9500
N1—C6	1.371 (3)	C24—C25	1.392 (4)
N3—C4	1.372 (3)	C24—H24	0.9500
N3—C2	1.377 (3)	C25—C26	1.380 (4)
N3—H3	0.8800	C25—H25	0.9500
N11—C12	1.344 (3)	C26—C27	1.388 (3)
N11—C16	1.357 (3)	C26—H26	0.9500
N22—C23	1.343 (3)	C27—C28	1.467 (3)
N22—C27	1.366 (3)	C28—C29	1.401 (3)
C1—C5	1.499 (3)	C28—C33	1.409 (3)
C1—H1A	0.9800	C29—C30	1.380 (4)
C1—H1B	0.9800	C29—H29	0.9500
C1—H1C	0.9800	C30—C31	1.384 (4)
C4—C5	1.437 (3)	C30—H30	0.9500
C5—C6	1.361 (3)	C31—C32	1.389 (4)
C6—H6	0.9500	C31—H31	0.9500
C12—C13	1.383 (4)	C32—C33	1.398 (3)
C12—H12	0.9500	C32—H32	0.9500
C13—C14	1.377 (4)	C51—H51A	0.9800
C13—H13	0.9500	C51—H51B	0.9800
C14—C15	1.383 (4)	C51—H51C	0.9800
C14—H14	0.9500	C61—H61A	0.9800
C15—C16	1.386 (4)	C61—H61B	0.9800
C15—H15	0.9500	C61—H61C	0.9800
			
C22—Rh1—C33	86.35 (9)	C18—C17—C22	121.0 (2)
C22—Rh1—N11	81.77 (9)	C18—C17—C16	123.4 (2)
C33—Rh1—N11	92.25 (8)	C22—C17—C16	115.6 (2)
C22—Rh1—N22	94.40 (9)	C19—C18—C17	119.8 (2)
C33—Rh1—N22	81.20 (9)	C19—C18—H18	120.1
N11—Rh1—N22	172.65 (7)	C17—C18—H18	120.1
C22—Rh1—O51	174.82 (8)	C18—C19—C20	120.0 (2)
C33—Rh1—O51	92.39 (8)	C18—C19—H19	120.0
N11—Rh1—O51	93.27 (7)	C20—C19—H19	120.0
N22—Rh1—O51	90.36 (7)	C19—C20—C21	120.2 (2)
C22—Rh1—N1	91.88 (8)	C19—C20—H20	119.9
C33—Rh1—N1	177.45 (8)	C21—C20—H20	119.9
N11—Rh1—N1	89.31 (7)	C22—C21—C20	121.0 (2)
N22—Rh1—N1	97.12 (7)	C22—C21—H21	119.5
O51—Rh1—N1	89.54 (6)	C20—C21—H21	119.5
C61—O61—H2	109.5	C21—C22—C17	117.9 (2)
C51—O51—Rh1	122.08 (16)	C21—C22—Rh1	128.13 (18)
C51—O51—H1	106 (2)	C17—C22—Rh1	113.87 (17)
Rh1—O51—H1	97 (2)	N22—C23—C24	122.5 (2)
C2—N1—C6	115.77 (19)	N22—C23—H23	118.7
C2—N1—Rh1	124.30 (15)	C24—C23—H23	118.7
C6—N1—Rh1	119.75 (15)	C23—C24—C25	118.8 (3)
C4—N3—C2	126.3 (2)	C23—C24—H24	120.6
C4—N3—H3	116.9	C25—C24—H24	120.6
C2—N3—H3	116.9	C26—C25—C24	119.2 (2)
C12—N11—C16	120.0 (2)	C26—C25—H25	120.4
C12—N11—Rh1	124.73 (17)	C24—C25—H25	120.4
C16—N11—Rh1	115.20 (15)	C25—C26—C27	119.8 (2)
C23—N22—C27	119.4 (2)	C25—C26—H26	120.1
C23—N22—Rh1	125.19 (16)	C27—C26—H26	120.1
C27—N22—Rh1	115.39 (15)	N22—C27—C26	120.3 (2)
C5—C1—H1A	109.5	N22—C27—C28	113.9 (2)
C5—C1—H1B	109.5	C26—C27—C28	125.8 (2)
H1A—C1—H1B	109.5	C29—C28—C33	121.0 (2)
C5—C1—H1C	109.5	C29—C28—C27	123.8 (2)
H1A—C1—H1C	109.5	C33—C28—C27	115.3 (2)
H1B—C1—H1C	109.5	C30—C29—C28	119.8 (2)
O2—C2—N1	123.4 (2)	C30—C29—H29	120.1
O2—C2—N3	117.1 (2)	C28—C29—H29	120.1
N1—C2—N3	119.6 (2)	C29—C30—C31	120.0 (2)
O4—C4—N3	119.6 (2)	C29—C30—H30	120.0
O4—C4—C5	126.4 (2)	C31—C30—H30	120.0
N3—C4—C5	113.9 (2)	C30—C31—C32	120.6 (2)
C6—C5—C4	117.4 (2)	C30—C31—H31	119.7
C6—C5—C1	123.0 (2)	C32—C31—H31	119.7
C4—C5—C1	119.6 (2)	C31—C32—C33	120.9 (2)
C5—C6—N1	127.0 (2)	C31—C32—H32	119.5
C5—C6—H6	116.5	C33—C32—H32	119.5
N1—C6—H6	116.5	C32—C33—C28	117.7 (2)
N11—C12—C13	121.6 (2)	C32—C33—Rh1	128.05 (18)
N11—C12—H12	119.2	C28—C33—Rh1	114.24 (16)
C13—C12—H12	119.2	O51—C51—H51A	109.5
C14—C13—C12	119.0 (3)	O51—C51—H51B	109.5
C14—C13—H13	120.5	H51A—C51—H51B	109.5
C12—C13—H13	120.5	O51—C51—H51C	109.5
C13—C14—C15	119.3 (3)	H51A—C51—H51C	109.5
C13—C14—H14	120.4	H51B—C51—H51C	109.5
C15—C14—H14	120.4	O61—C61—H61A	109.5
C14—C15—C16	119.9 (3)	O61—C61—H61B	109.5
C14—C15—H15	120.0	H61A—C61—H61B	109.5
C16—C15—H15	120.0	O61—C61—H61C	109.5
N11—C16—C15	120.1 (2)	H61A—C61—H61C	109.5
N11—C16—C17	113.5 (2)	H61B—C61—H61C	109.5
C15—C16—C17	126.4 (2)		
			
C6—N1—C2—O2	−175.8 (3)	C18—C19—C20—C21	−0.2 (4)
Rh1—N1—C2—O2	−0.7 (4)	C19—C20—C21—C22	0.5 (4)
C6—N1—C2—N3	4.4 (3)	C20—C21—C22—C17	−0.6 (3)
Rh1—N1—C2—N3	179.45 (17)	C20—C21—C22—Rh1	175.86 (18)
C4—N3—C2—O2	176.3 (2)	C18—C17—C22—C21	0.4 (3)
C4—N3—C2—N1	−3.8 (4)	C16—C17—C22—C21	178.3 (2)
C2—N3—C4—O4	−178.9 (2)	C18—C17—C22—Rh1	−176.57 (19)
C2—N3—C4—C5	0.3 (4)	C16—C17—C22—Rh1	1.4 (3)
O4—C4—C5—C6	−178.7 (2)	C27—N22—C23—C24	−0.8 (4)
N3—C4—C5—C6	2.1 (3)	Rh1—N22—C23—C24	−178.6 (2)
O4—C4—C5—C1	2.1 (4)	N22—C23—C24—C25	0.9 (5)
N3—C4—C5—C1	−177.2 (2)	C23—C24—C25—C26	0.0 (5)
C4—C5—C6—N1	−1.4 (4)	C24—C25—C26—C27	−1.0 (5)
C1—C5—C6—N1	177.9 (3)	C23—N22—C27—C26	−0.2 (4)
C2—N1—C6—C5	−2.0 (4)	Rh1—N22—C27—C26	177.76 (19)
Rh1—N1—C6—C5	−177.3 (2)	C23—N22—C27—C28	−179.4 (2)
C16—N11—C12—C13	−0.3 (4)	Rh1—N22—C27—C28	−1.5 (3)
Rh1—N11—C12—C13	176.5 (2)	C25—C26—C27—N22	1.1 (4)
N11—C12—C13—C14	−0.2 (4)	C25—C26—C27—C28	−179.8 (3)
C12—C13—C14—C15	0.0 (4)	N22—C27—C28—C29	−177.8 (2)
C13—C14—C15—C16	0.7 (5)	C26—C27—C28—C29	3.0 (4)
C12—N11—C16—C15	1.0 (3)	N22—C27—C28—C33	1.2 (3)
Rh1—N11—C16—C15	−176.1 (2)	C26—C27—C28—C33	−178.0 (2)
C12—N11—C16—C17	179.9 (2)	C33—C28—C29—C30	0.2 (4)
Rh1—N11—C16—C17	2.8 (3)	C27—C28—C29—C30	179.1 (2)
C14—C15—C16—N11	−1.2 (4)	C28—C29—C30—C31	−0.1 (4)
C14—C15—C16—C17	−180.0 (3)	C29—C30—C31—C32	0.2 (4)
N11—C16—C17—C18	175.1 (2)	C30—C31—C32—C33	−0.5 (4)
C15—C16—C17—C18	−6.0 (4)	C31—C32—C33—C28	0.6 (4)
N11—C16—C17—C22	−2.8 (3)	C31—C32—C33—Rh1	−178.35 (19)
C15—C16—C17—C22	176.1 (2)	C29—C28—C33—C32	−0.5 (3)
C22—C17—C18—C19	−0.1 (4)	C27—C28—C33—C32	−179.4 (2)
C16—C17—C18—C19	−177.9 (2)	C29—C28—C33—Rh1	178.66 (19)
C17—C18—C19—C20	0.0 (4)	C27—C28—C33—Rh1	−0.3 (3)

### (2) (OC-6-42)-Methanol(5-methyl-2,4-dioxo-1,2,3,4-tetrahydropyrimidin-1-ido-κN1)bis[2-(pyridin-2-yl)phenyl-κ2N,C1]rhodium(III) Hydrogen-bond geometry (Å, º)

**Table:**

*D*—H···*A*	*D*—H	H···*A*	*D*···*A*	*D*—H···*A*
O51—H1···O2	0.84 (2)	1.69 (2)	2.527 (3)	170 (3)
N3—H3···O2^i^	0.88	1.97	2.844 (3)	173
O61—H2···O4^ii^	0.84	2.01	2.802 (5)	157

Symmetry codes: (i) −*x*, −*y*+1, −*z*+1; (ii) −*x*+1/2, *y*−1/2, *z*.

### (3) (OC-6-42)-Ethanol(5-methyl-2,4-dioxo-1,2,3,4-tetrahydropyrimidin-1-ido-κN1)bis[2-(pyridin-2-yl)phenyl-κ2N,C1]rhodium(III) Crystal data

**Table:**

[Rh(C_11_H_8_N)_2_(C_5_H_5_N_2_O_2_)(C_2_H_6_O)]·C_2_H_6_O	*D*_x_ = 1.479 Mg m^−^^3^
*M**_r_* = 628.52	Mo *K*α radiation, λ = 0.71075 Å
Orthorhombic, *P**b**c**a*	Cell parameters from 41739 reflections
*a* = 11.1082 (5) Å	θ = 3.1–27.6°
*b* = 15.5556 (6) Å	µ = 0.65 mm^−^^1^
*c* = 32.6747 (15) Å	*T* = 192 K
*V* = 5646.0 (4) Å^3^	Block, yellow
*Z* = 8	0.30 × 0.20 × 0.20 mm
*F*(000) = 2592	

### (3) (OC-6-42)-Ethanol(5-methyl-2,4-dioxo-1,2,3,4-tetrahydropyrimidin-1-ido-κN1)bis[2-(pyridin-2-yl)phenyl-κ2N,C1]rhodium(III) Data collection

**Table:**

Rigaku R-AXIS RAPID diffractometer	6470 independent reflections
Radiation source: fine-focus sealed tube	5886 reflections with *I* > 2σ(*I*)
Graphite monochromator	*R*_int_ = 0.030
Detector resolution: 10.000 pixels mm^-1^	θ_max_ = 27.5°, θ_min_ = 3.1°
ω scans	*h* = −14→14
Absorption correction: numerical (NUMABS; Rigaku, 1999)	*k* = −20→20
*T*_min_ = 0.829, *T*_max_ = 0.881	*l* = −42→40
52659 measured reflections	

### (3) (OC-6-42)-Ethanol(5-methyl-2,4-dioxo-1,2,3,4-tetrahydropyrimidin-1-ido-κN1)bis[2-(pyridin-2-yl)phenyl-κ2N,C1]rhodium(III) Refinement

**Table:**

Refinement on *F*^2^	Primary atom site location: structure-invariant direct methods
Least-squares matrix: full	Secondary atom site location: difference Fourier map
*R*[*F*^2^ > 2σ(*F*^2^)] = 0.029	Hydrogen site location: inferred from neighbouring sites
*wR*(*F*^2^) = 0.076	H atoms treated by a mixture of independent and constrained refinement
*S* = 1.07	*w* = 1/[σ^2^(*F*_o_^2^) + (0.0365*P*)^2^ + 4.0646*P*] where *P* = (*F*_o_^2^ + 2*F*_c_^2^)/3
6470 reflections	(Δ/σ)_max_ = 0.002
370 parameters	Δρ_max_ = 1.08 e Å^−^^3^
2 restraints	Δρ_min_ = −0.40 e Å^−^^3^

### (3) (OC-6-42)-Ethanol(5-methyl-2,4-dioxo-1,2,3,4-tetrahydropyrimidin-1-ido-κN1)bis[2-(pyridin-2-yl)phenyl-κ2N,C1]rhodium(III) Special details

**Table:**

Experimental. The ^1^H NMR spectrum of 3 in CDCl_3_ at 22 °C: δ 1.53 (s, 3H, Hthym C*H*_3_), 6.17 (d, *J* = 7.7 Hz, 2H, ppy), 6.42 (s, 1H, Hthym C^6^-*H*), 6.81 (t, *J* = 7.4 Hz, 2H, ppy), 6.95 (t, *J* = 7.40 Hz, 2H, ppy), 7.28–7.30 (m, 2H, ppy), 7.58–7.61 (m, 2H, ppy), 7.88–7.92 (m, 4H, ppy), 8.10 (s, 1H, Hthym N^3^-*H*), 8.59 (d, *J* = 5.5 Hz, 1H, ppy) and 9.01 (d, *J* = 5.8 Hz, 1H, ppy).
Geometry. All esds (except the esd in the dihedral angle between two l.s. planes) are estimated using the full covariance matrix. The cell esds are taken into account individually in the estimation of esds in distances, angles and torsion angles; correlations between esds in cell parameters are only used when they are defined by crystal symmetry. An approximate (isotropic) treatment of cell esds is used for estimating esds involving l.s. planes.

### (3) (OC-6-42)-Ethanol(5-methyl-2,4-dioxo-1,2,3,4-tetrahydropyrimidin-1-ido-κN1)bis[2-(pyridin-2-yl)phenyl-κ2N,C1]rhodium(III) Fractional atomic coordinates and isotropic or equivalent isotropic displacement parameters (Å^2^)

**Table:**

	*x*	*y*	*z*	*U*_iso_*/*U*_eq_	
Rh1	0.21339 (2)	0.02045 (2)	0.63615 (2)	0.02373 (6)	
O2	0.04210 (14)	0.02427 (10)	0.54944 (5)	0.0446 (4)	
O4	0.18318 (15)	−0.19254 (10)	0.47134 (4)	0.0420 (3)	
O51	0.09395 (13)	0.11923 (9)	0.60966 (4)	0.0350 (3)	
H1	0.065 (2)	0.0932 (14)	0.5896 (5)	0.042*	
O61	0.2437 (4)	0.16196 (16)	0.42791 (10)	0.1286 (13)	
H2	0.286 (4)	0.200 (3)	0.4388 (16)	0.154*	
N1	0.20026 (14)	−0.05157 (11)	0.57654 (5)	0.0281 (3)	
N3	0.11666 (15)	−0.08594 (11)	0.51266 (5)	0.0332 (4)	
H3	0.0599	−0.0752	0.4946	0.040*	
N11	0.36121 (14)	0.08209 (10)	0.61499 (5)	0.0284 (3)	
N22	0.06964 (15)	−0.03762 (10)	0.66331 (5)	0.0303 (3)	
C1	0.3788 (2)	−0.23404 (14)	0.52698 (7)	0.0427 (5)	
H1A	0.4369	−0.2336	0.5496	0.051*	
H1B	0.4207	−0.2223	0.5012	0.051*	
H1C	0.3400	−0.2905	0.5255	0.051*	
C2	0.11859 (17)	−0.03524 (13)	0.54715 (6)	0.0308 (4)	
C4	0.19492 (17)	−0.15193 (13)	0.50373 (6)	0.0313 (4)	
C5	0.28503 (17)	−0.16615 (12)	0.53424 (6)	0.0305 (4)	
C6	0.28117 (16)	−0.11607 (13)	0.56826 (6)	0.0300 (4)	
H6	0.3407	−0.1268	0.5885	0.036*	
C12	0.35950 (19)	0.15538 (13)	0.59301 (6)	0.0368 (4)	
H12	0.2841	0.1797	0.5856	0.044*	
C13	0.4633 (2)	0.19605 (15)	0.58094 (7)	0.0445 (5)	
H13	0.4598	0.2478	0.5655	0.053*	
C14	0.5724 (2)	0.16073 (16)	0.59152 (8)	0.0490 (6)	
H14	0.6454	0.1877	0.5834	0.059*	
C15	0.57473 (19)	0.08549 (15)	0.61410 (7)	0.0430 (5)	
H15	0.6496	0.0605	0.6216	0.052*	
C16	0.46783 (17)	0.04653 (13)	0.62583 (6)	0.0310 (4)	
C17	0.45550 (17)	−0.03176 (12)	0.65058 (6)	0.0298 (4)	
C18	0.55323 (19)	−0.07910 (14)	0.66478 (7)	0.0390 (5)	
H18	0.6329	−0.0618	0.6582	0.047*	
C19	0.5340 (2)	−0.15135 (14)	0.68842 (7)	0.0421 (5)	
H19	0.6006	−0.1835	0.6984	0.050*	
C20	0.4182 (2)	−0.17709 (13)	0.69765 (6)	0.0376 (4)	
H20	0.4055	−0.2267	0.7140	0.045*	
C21	0.32003 (18)	−0.13088 (12)	0.68316 (6)	0.0317 (4)	
H21	0.2408	−0.1495	0.6895	0.038*	
C22	0.33663 (16)	−0.05717 (12)	0.65930 (5)	0.0263 (3)	
C23	−0.00383 (19)	−0.09457 (14)	0.64515 (6)	0.0373 (4)	
H23	0.0148	−0.1136	0.6183	0.045*	
C24	−0.1053 (2)	−0.12629 (16)	0.66412 (7)	0.0475 (5)	
H24	−0.1558	−0.1666	0.6507	0.057*	
C25	−0.1317 (2)	−0.09808 (18)	0.70328 (7)	0.0500 (6)	
H25	−0.2011	−0.1190	0.7171	0.060*	
C26	−0.0573 (2)	−0.03967 (16)	0.72216 (7)	0.0428 (5)	
H26	−0.0752	−0.0199	0.7490	0.051*	
C27	0.04446 (18)	−0.00956 (13)	0.70185 (6)	0.0319 (4)	
C28	0.13082 (18)	0.05356 (13)	0.71778 (6)	0.0315 (4)	
C29	0.1252 (2)	0.08784 (14)	0.75730 (6)	0.0410 (5)	
H29	0.0629	0.0706	0.7755	0.049*	
C30	0.2101 (2)	0.14662 (17)	0.76979 (7)	0.0471 (6)	
H30	0.2067	0.1698	0.7967	0.057*	
C31	0.3000 (2)	0.17192 (15)	0.74343 (7)	0.0455 (5)	
H31	0.3587	0.2123	0.7523	0.055*	
C32	0.30553 (18)	0.13867 (13)	0.70386 (6)	0.0357 (4)	
H32	0.3674	0.1573	0.6859	0.043*	
C33	0.22157 (16)	0.07850 (12)	0.69031 (6)	0.0284 (4)	
C51	0.0044 (2)	0.16798 (18)	0.62979 (8)	0.0531 (6)	
H51A	0.0037	0.2267	0.6181	0.064*	
H51B	0.0261	0.1730	0.6591	0.064*	
C52	−0.1159 (2)	0.1322 (2)	0.62678 (9)	0.0660 (8)	
H52A	−0.1196	0.0782	0.6422	0.079*	
H52B	−0.1351	0.1212	0.5980	0.079*	
H52C	−0.1741	0.1731	0.6381	0.079*	
C61	0.2473 (4)	0.0866 (2)	0.44860 (13)	0.0826 (10)	
H61A	0.2355	0.0396	0.4286	0.099*	
H61B	0.1776	0.0854	0.4675	0.099*	
C62	0.3536 (3)	0.0669 (2)	0.47211 (13)	0.0849 (10)	
H62A	0.3677	0.1127	0.4922	0.102*	
H62B	0.4231	0.0626	0.4537	0.102*	
H62C	0.3423	0.0121	0.4864	0.102*	

### (3) (OC-6-42)-Ethanol(5-methyl-2,4-dioxo-1,2,3,4-tetrahydropyrimidin-1-ido-κN1)bis[2-(pyridin-2-yl)phenyl-κ2N,C1]rhodium(III) Atomic displacement parameters (Å^2^)

**Table:**

	*U*^11^	*U*^22^	*U*^33^	*U*^12^	*U*^13^	*U*^23^
Rh1	0.02103 (8)	0.02848 (9)	0.02167 (8)	−0.00084 (5)	−0.00246 (5)	−0.00128 (5)
O2	0.0414 (8)	0.0577 (10)	0.0347 (8)	0.0210 (7)	−0.0152 (7)	−0.0141 (7)
O4	0.0502 (9)	0.0446 (8)	0.0313 (7)	0.0013 (7)	−0.0040 (6)	−0.0118 (6)
O51	0.0326 (7)	0.0364 (7)	0.0359 (7)	0.0067 (6)	−0.0090 (6)	−0.0056 (6)
O61	0.227 (4)	0.0573 (14)	0.101 (2)	0.0135 (19)	−0.095 (2)	−0.0207 (14)
N1	0.0277 (8)	0.0331 (8)	0.0236 (7)	0.0015 (6)	−0.0042 (6)	−0.0021 (6)
N3	0.0317 (8)	0.0428 (9)	0.0251 (7)	0.0027 (7)	−0.0081 (6)	−0.0056 (7)
N11	0.0260 (7)	0.0333 (8)	0.0260 (7)	−0.0025 (6)	−0.0026 (6)	0.0011 (6)
N22	0.0264 (8)	0.0360 (8)	0.0285 (8)	−0.0027 (7)	−0.0026 (6)	0.0006 (6)
C1	0.0490 (13)	0.0445 (12)	0.0346 (10)	0.0128 (10)	0.0014 (9)	−0.0023 (9)
C2	0.0287 (9)	0.0379 (10)	0.0258 (9)	0.0011 (8)	−0.0031 (7)	−0.0039 (7)
C4	0.0338 (10)	0.0332 (9)	0.0269 (9)	−0.0039 (8)	0.0016 (7)	−0.0011 (7)
C5	0.0338 (10)	0.0308 (9)	0.0269 (9)	0.0012 (8)	0.0018 (7)	0.0015 (7)
C6	0.0302 (10)	0.0352 (9)	0.0247 (8)	0.0015 (8)	−0.0028 (7)	0.0006 (7)
C12	0.0361 (10)	0.0391 (10)	0.0351 (10)	−0.0013 (9)	−0.0034 (8)	0.0078 (8)
C13	0.0453 (13)	0.0424 (11)	0.0458 (12)	−0.0057 (10)	0.0020 (10)	0.0147 (10)
C14	0.0367 (12)	0.0525 (13)	0.0579 (14)	−0.0114 (10)	0.0062 (10)	0.0125 (11)
C15	0.0275 (10)	0.0492 (12)	0.0521 (13)	−0.0019 (9)	0.0012 (9)	0.0080 (10)
C16	0.0269 (9)	0.0353 (9)	0.0308 (9)	−0.0014 (8)	−0.0013 (7)	−0.0008 (8)
C17	0.0266 (9)	0.0325 (9)	0.0304 (9)	−0.0005 (7)	−0.0041 (7)	−0.0015 (7)
C18	0.0274 (10)	0.0408 (11)	0.0487 (12)	0.0020 (9)	−0.0060 (9)	0.0024 (9)
C19	0.0381 (11)	0.0394 (11)	0.0487 (12)	0.0062 (9)	−0.0111 (9)	0.0050 (9)
C20	0.0487 (12)	0.0306 (9)	0.0335 (10)	0.0024 (9)	−0.0035 (9)	0.0030 (8)
C21	0.0348 (10)	0.0331 (9)	0.0271 (9)	−0.0017 (8)	−0.0004 (8)	−0.0006 (7)
C22	0.0275 (9)	0.0298 (8)	0.0217 (8)	0.0003 (7)	−0.0044 (7)	−0.0033 (7)
C23	0.0329 (10)	0.0448 (11)	0.0343 (10)	−0.0078 (9)	−0.0016 (8)	−0.0031 (9)
C24	0.0382 (12)	0.0567 (14)	0.0475 (12)	−0.0181 (11)	−0.0037 (10)	0.0005 (11)
C25	0.0351 (11)	0.0698 (16)	0.0450 (12)	−0.0142 (11)	0.0028 (10)	0.0105 (11)
C26	0.0367 (11)	0.0573 (13)	0.0344 (10)	−0.0038 (10)	0.0049 (9)	0.0038 (9)
C27	0.0290 (9)	0.0395 (10)	0.0272 (9)	0.0011 (8)	−0.0001 (7)	0.0024 (8)
C28	0.0319 (10)	0.0365 (9)	0.0262 (9)	0.0039 (8)	−0.0026 (7)	−0.0002 (8)
C29	0.0464 (12)	0.0499 (12)	0.0266 (9)	0.0052 (10)	0.0011 (9)	−0.0032 (8)
C30	0.0555 (14)	0.0558 (14)	0.0300 (10)	0.0076 (11)	−0.0072 (9)	−0.0123 (10)
C31	0.0458 (13)	0.0454 (12)	0.0455 (13)	−0.0004 (10)	−0.0135 (10)	−0.0160 (10)
C32	0.0325 (10)	0.0374 (10)	0.0373 (11)	−0.0006 (8)	−0.0039 (8)	−0.0061 (8)
C33	0.0271 (9)	0.0311 (9)	0.0269 (8)	0.0035 (7)	−0.0050 (7)	−0.0034 (7)
C51	0.0538 (15)	0.0620 (15)	0.0435 (12)	0.0242 (13)	−0.0116 (11)	−0.0180 (11)
C52	0.0464 (15)	0.099 (2)	0.0528 (15)	0.0173 (15)	0.0058 (12)	0.0035 (15)
C61	0.086 (2)	0.0521 (17)	0.110 (3)	−0.0008 (17)	−0.019 (2)	−0.0211 (18)
C62	0.090 (3)	0.0581 (18)	0.106 (3)	−0.0073 (18)	−0.012 (2)	0.0058 (18)

### (3) (OC-6-42)-Ethanol(5-methyl-2,4-dioxo-1,2,3,4-tetrahydropyrimidin-1-ido-κN1)bis[2-(pyridin-2-yl)phenyl-κ2N,C1]rhodium(III) Geometric parameters (Å, º)

**Table:**

Rh1—C22	1.9760 (18)	C18—C19	1.380 (3)
Rh1—C33	1.9890 (18)	C18—H18	0.9500
Rh1—N11	2.0232 (15)	C19—C20	1.381 (3)
Rh1—N22	2.0381 (16)	C19—H19	0.9500
Rh1—O51	2.2068 (14)	C20—C21	1.389 (3)
Rh1—N1	2.2516 (15)	C20—H20	0.9500
O2—C2	1.259 (2)	C21—C22	1.399 (3)
O4—C4	1.240 (2)	C21—H21	0.9500
O51—C51	1.414 (3)	C23—C24	1.377 (3)
O51—H1	0.833 (10)	C23—H23	0.9500
O61—C61	1.354 (4)	C24—C25	1.384 (3)
O61—H2	0.836 (10)	C24—H24	0.9500
N1—C2	1.345 (2)	C25—C26	1.375 (3)
N1—C6	1.374 (2)	C25—H25	0.9500
N3—C2	1.376 (2)	C26—C27	1.392 (3)
N3—C4	1.376 (3)	C26—H26	0.9500
N3—H3	0.8800	C27—C28	1.468 (3)
N11—C12	1.347 (2)	C28—C29	1.399 (3)
N11—C16	1.354 (2)	C28—C33	1.404 (3)
N22—C23	1.343 (3)	C29—C30	1.376 (3)
N22—C27	1.362 (3)	C29—H29	0.9500
C1—C5	1.502 (3)	C30—C31	1.377 (4)
C1—H1A	0.9800	C30—H30	0.9500
C1—H1B	0.9800	C31—C32	1.394 (3)
C1—H1C	0.9800	C31—H31	0.9500
C4—C5	1.430 (3)	C32—C33	1.393 (3)
C5—C6	1.358 (3)	C32—H32	0.9500
C6—H6	0.9500	C51—C52	1.450 (4)
C12—C13	1.373 (3)	C51—H51A	0.9900
C12—H12	0.9500	C51—H51B	0.9900
C13—C14	1.375 (3)	C52—H52A	0.9800
C13—H13	0.9500	C52—H52B	0.9800
C14—C15	1.384 (3)	C52—H52C	0.9800
C14—H14	0.9500	C61—C62	1.442 (5)
C15—C16	1.387 (3)	C61—H61A	0.9900
C15—H15	0.9500	C61—H61B	0.9900
C16—C17	1.468 (3)	C62—H62A	0.9800
C17—C18	1.391 (3)	C62—H62B	0.9800
C17—C22	1.407 (3)	C62—H62C	0.9800
			
C22—Rh1—C33	84.55 (7)	C18—C19—C20	120.18 (19)
C22—Rh1—N11	81.84 (7)	C18—C19—H19	119.9
C33—Rh1—N11	92.97 (7)	C20—C19—H19	119.9
C22—Rh1—N22	96.04 (7)	C19—C20—C21	120.44 (19)
C33—Rh1—N22	81.34 (7)	C19—C20—H20	119.8
N11—Rh1—N22	174.11 (6)	C21—C20—H20	119.8
C22—Rh1—O51	172.81 (7)	C20—C21—C22	120.71 (19)
C33—Rh1—O51	93.46 (6)	C20—C21—H21	119.6
N11—Rh1—O51	91.38 (6)	C22—C21—H21	119.6
N22—Rh1—O51	90.48 (6)	C21—C22—C17	117.82 (17)
C22—Rh1—N1	94.13 (6)	C21—C22—Rh1	128.53 (14)
C33—Rh1—N1	176.92 (7)	C17—C22—Rh1	113.63 (13)
N11—Rh1—N1	89.59 (6)	N22—C23—C24	122.3 (2)
N22—Rh1—N1	96.06 (6)	N22—C23—H23	118.8
O51—Rh1—N1	88.18 (5)	C24—C23—H23	118.8
C51—O51—Rh1	127.90 (14)	C23—C24—C25	118.4 (2)
C51—O51—H1	110.9 (17)	C23—C24—H24	120.8
Rh1—O51—H1	101.5 (17)	C25—C24—H24	120.8
C61—O61—H2	113 (4)	C26—C25—C24	119.8 (2)
C2—N1—C6	116.01 (16)	C26—C25—H25	120.1
C2—N1—Rh1	124.55 (13)	C24—C25—H25	120.1
C6—N1—Rh1	119.42 (12)	C25—C26—C27	119.8 (2)
C2—N3—C4	126.26 (16)	C25—C26—H26	120.1
C2—N3—H3	116.9	C27—C26—H26	120.1
C4—N3—H3	116.9	N22—C27—C26	119.99 (19)
C12—N11—C16	119.82 (17)	N22—C27—C28	114.08 (17)
C12—N11—Rh1	124.86 (13)	C26—C27—C28	125.92 (19)
C16—N11—Rh1	115.27 (12)	C29—C28—C33	121.12 (19)
C23—N22—C27	119.70 (17)	C29—C28—C27	123.58 (19)
C23—N22—Rh1	125.17 (14)	C33—C28—C27	115.30 (16)
C27—N22—Rh1	114.94 (13)	C30—C29—C28	119.8 (2)
C5—C1—H1A	109.5	C30—C29—H29	120.1
C5—C1—H1B	109.5	C28—C29—H29	120.1
H1A—C1—H1B	109.5	C29—C30—C31	120.1 (2)
C5—C1—H1C	109.5	C29—C30—H30	119.9
H1A—C1—H1C	109.5	C31—C30—H30	119.9
H1B—C1—H1C	109.5	C30—C31—C32	120.4 (2)
O2—C2—N1	123.48 (17)	C30—C31—H31	119.8
O2—C2—N3	117.38 (17)	C32—C31—H31	119.8
N1—C2—N3	119.14 (17)	C33—C32—C31	121.0 (2)
O4—C4—N3	119.64 (18)	C33—C32—H32	119.5
O4—C4—C5	126.16 (19)	C31—C32—H32	119.5
N3—C4—C5	114.19 (17)	C32—C33—C28	117.59 (18)
C6—C5—C4	117.37 (17)	C32—C33—Rh1	128.18 (15)
C6—C5—C1	123.66 (18)	C28—C33—Rh1	114.23 (13)
C4—C5—C1	118.96 (18)	O51—C51—C52	114.3 (2)
C5—C6—N1	126.91 (17)	O51—C51—H51A	108.7
C5—C6—H6	116.5	C52—C51—H51A	108.7
N1—C6—H6	116.5	O51—C51—H51B	108.7
N11—C12—C13	122.1 (2)	C52—C51—H51B	108.7
N11—C12—H12	119.0	H51A—C51—H51B	107.6
C13—C12—H12	119.0	C51—C52—H52A	109.5
C12—C13—C14	118.9 (2)	C51—C52—H52B	109.5
C12—C13—H13	120.5	H52A—C52—H52B	109.5
C14—C13—H13	120.5	C51—C52—H52C	109.5
C13—C14—C15	119.3 (2)	H52A—C52—H52C	109.5
C13—C14—H14	120.4	H52B—C52—H52C	109.5
C15—C14—H14	120.4	O61—C61—C62	118.3 (3)
C14—C15—C16	120.0 (2)	O61—C61—H61A	107.7
C14—C15—H15	120.0	C62—C61—H61A	107.7
C16—C15—H15	120.0	O61—C61—H61B	107.7
N11—C16—C15	119.87 (18)	C62—C61—H61B	107.7
N11—C16—C17	113.66 (16)	H61A—C61—H61B	107.1
C15—C16—C17	126.47 (18)	C61—C62—H62A	109.5
C18—C17—C22	121.06 (18)	C61—C62—H62B	109.5
C18—C17—C16	123.36 (18)	H62A—C62—H62B	109.5
C22—C17—C16	115.59 (16)	C61—C62—H62C	109.5
C19—C18—C17	119.8 (2)	H62A—C62—H62C	109.5
C19—C18—H18	120.1	H62B—C62—H62C	109.5
C17—C18—H18	120.1		
			
C6—N1—C2—O2	−176.78 (19)	C19—C20—C21—C22	−0.6 (3)
Rh1—N1—C2—O2	2.0 (3)	C20—C21—C22—C17	0.0 (3)
C6—N1—C2—N3	3.7 (3)	C20—C21—C22—Rh1	−178.63 (14)
Rh1—N1—C2—N3	−177.51 (13)	C18—C17—C22—C21	0.9 (3)
C4—N3—C2—O2	177.57 (19)	C16—C17—C22—C21	−179.34 (16)
C4—N3—C2—N1	−2.9 (3)	C18—C17—C22—Rh1	179.69 (16)
C2—N3—C4—O4	−179.81 (19)	C16—C17—C22—Rh1	−0.5 (2)
C2—N3—C4—C5	−0.1 (3)	C27—N22—C23—C24	0.0 (3)
O4—C4—C5—C6	−178.4 (2)	Rh1—N22—C23—C24	−174.60 (17)
N3—C4—C5—C6	1.9 (3)	N22—C23—C24—C25	0.1 (4)
O4—C4—C5—C1	2.3 (3)	C23—C24—C25—C26	0.1 (4)
N3—C4—C5—C1	−177.40 (18)	C24—C25—C26—C27	−0.2 (4)
C4—C5—C6—N1	−1.0 (3)	C23—N22—C27—C26	−0.2 (3)
C1—C5—C6—N1	178.31 (19)	Rh1—N22—C27—C26	174.94 (16)
C2—N1—C6—C5	−1.9 (3)	C23—N22—C27—C28	−179.02 (18)
Rh1—N1—C6—C5	179.19 (16)	Rh1—N22—C27—C28	−3.9 (2)
C16—N11—C12—C13	0.1 (3)	C25—C26—C27—N22	0.3 (3)
Rh1—N11—C12—C13	177.28 (17)	C25—C26—C27—C28	179.0 (2)
N11—C12—C13—C14	0.2 (4)	N22—C27—C28—C29	−177.02 (19)
C12—C13—C14—C15	−0.2 (4)	C26—C27—C28—C29	4.3 (3)
C13—C14—C15—C16	0.0 (4)	N22—C27—C28—C33	3.2 (3)
C12—N11—C16—C15	−0.3 (3)	C26—C27—C28—C33	−175.5 (2)
Rh1—N11—C16—C15	−177.76 (16)	C33—C28—C29—C30	−0.4 (3)
C12—N11—C16—C17	178.46 (17)	C27—C28—C29—C30	179.8 (2)
Rh1—N11—C16—C17	1.0 (2)	C28—C29—C30—C31	0.3 (3)
C14—C15—C16—N11	0.3 (3)	C29—C30—C31—C32	0.3 (4)
C14—C15—C16—C17	−178.3 (2)	C30—C31—C32—C33	−0.9 (3)
N11—C16—C17—C18	179.47 (18)	C31—C32—C33—C28	0.7 (3)
C15—C16—C17—C18	−1.9 (3)	C31—C32—C33—Rh1	−178.48 (16)
N11—C16—C17—C22	−0.3 (3)	C29—C28—C33—C32	−0.1 (3)
C15—C16—C17—C22	178.3 (2)	C27—C28—C33—C32	179.67 (18)
C22—C17—C18—C19	−1.2 (3)	C29—C28—C33—Rh1	179.23 (16)
C16—C17—C18—C19	179.0 (2)	C27—C28—C33—Rh1	−1.0 (2)
C17—C18—C19—C20	0.6 (3)	Rh1—O51—C51—C52	−94.8 (2)
C18—C19—C20—C21	0.3 (3)		

### (3) (OC-6-42)-Ethanol(5-methyl-2,4-dioxo-1,2,3,4-tetrahydropyrimidin-1-ido-κN1)bis[2-(pyridin-2-yl)phenyl-κ2N,C1]rhodium(III) Hydrogen-bond geometry (Å, º)

**Table:**

*D*—H···*A*	*D*—H	H···*A*	*D*···*A*	*D*—H···*A*
O51—H1···O2	0.83 (1)	1.72 (2)	2.527 (2)	164 (2)
N3—H3···O2^i^	0.88	1.99	2.854 (2)	165
O61—H2···O4^ii^	0.84 (1)	2.01 (4)	2.792 (3)	156 (4)

Symmetry codes: (i) −*x*, −*y*, −*z*+1; (ii) −*x*+1/2, *y*+1/2, *z*.
